# Obesity alters oestrogen metabolism and contributes to pulmonary arterial hypertension

**DOI:** 10.1183/13993003.01524-2018

**Published:** 2019-06-13

**Authors:** Kirsty M. Mair, Katie Y. Harvey, Alasdair D. Henry, Dianne Z. Hillyard, Margaret Nilsen, Margaret R. MacLean

**Affiliations:** 1 Institute of Cardiovascular and Medical Sciences, College of Medical and Veterinary Science, University of Glasgow, Glasgow, UK; 2 Strathclyde Institute of Pharmacy and Biomedical Sciences, University of Strathclyde, Glasgow, UK

## Abstract

Obesity is a common comorbidity for pulmonary arterial hypertension (PAH). Additionally, oestrogen and its metabolites are risk factors for the development of PAH. Visceral adipose tissue (VAT) is a major site of oestrogen production; however, the influence of obesity-induced changes in oestrogen synthesis and metabolism on the development of PAH is unclear. To address this we investigated the effects of inhibiting oestrogen synthesis and metabolism on the development of pulmonary hypertension in male and female obese mice.

We depleted endogenous oestrogen in leptin-deficient (*ob*/*ob*) mice with the oestrogen inhibitor anastrozole (ANA) and determined the effects on the development of pulmonary hypertension, plasma oestradiol and urinary 16α-hydroxyestrone (16αOHE1). Oestrogen metabolism through cytochrome P450 1B1 (CYP1B1) was inhibited with 2,2′,4,6′-tetramethoxystilbene (TMS).

*ob*/*ob* mice spontaneously develop pulmonary hypertension, pulmonary vascular remodelling and increased reactive oxygen species production in the lung; these effects were attenuated by ANA. Oestradiol levels were decreased in obese male mice; however, VAT CYP1B1 and 16αOHE1 levels were increased. TMS also attenuated pulmonary hypertension in male *ob*/*ob* mice. Intra-thoracic fat from *ob*/*ob* mice and VAT conditioned media produce 16αOHE1 and can contribute to oxidative stress, effects that are attenuated by both ANA and TMS.

Obesity can induce pulmonary hypertension and changes in oestrogen metabolism, resulting in increased production of 16αOHE1 from VAT that contributes to oxidative stress. Oestrogen inhibitors are now in clinical trials for PAH. This study has translational consequences as it suggests that oestrogen inhibitors may be especially beneficial in treating obese individuals with PAH.

## Abstract 2

**Obesity is a risk factor in patients with PAH. This study suggests that this is due to altered oestrogen metabolism in adipose tissue. Inhibition of oestrogen production or metabolism may be of benefit to obese PAH patients.** http://ow.ly/2zW830of1fG

## Introduction

Obesity is a well-recognised independent cardiovascular risk factor and is a comorbidity to pulmonary arterial hypertension (PAH) [1]. In the REVEAL (Registry to EValuate Early And Long-term PAH disease management) registry, 32% of patients with PAH were classified as obese at enrolment [2]. This registry reports a higher prevalence of overweight and obese individuals among those with the idiopathic form of PAH [1]. Obesity-related pulmonary hypertension can occur as a result of hypoventilation and hypoxia due to the increased mechanical load of excess body fat [3]. However, the metabolic and inflammatory disturbances that occur during obesity may play a role in the development of PAH.

Adipose tissue expresses high levels of the oestrogen-synthesising enzyme aromatase and in obesity can become a major source of oestrogen production [4]. Interestingly, adipose tissue expresses high levels of cytochrome P450 1B1 (CYP1B1), an enzyme involved in oestrogen metabolism [5]. CYP1B1 plays a role in clinical PAH, with CYP1B1 highly expressed in pulmonary artery lesions of PAH patients [6–8]. Furthermore, CYP1B1 single nucleotide polymorphisms have been identified that have significant association with right ventricular ejection fraction and oestrogen metabolism [9]. These are in tight linkage disequilibrium with single nucleotide polymorphisms associated with pulmonary hypertension and oncogenesis [9].

The oestrogen metabolite 16α-hydroxyestrone (16αOHE1) is formed *via* CYP1B1 and has been implicated in PAH. 16αOHE1 is a potent mitogen in pulmonary artery smooth muscle cells (PASMCs), acting in a reactive oxygen species (ROS)-dependent manner with little effect on smooth muscle cells from the systemic circulation [10]. Furthermore, when administered to mice, 16αOHE1 results in the development of a pulmonary hypertension phenotype [8].

Growing evidence suggests that endogenous oestrogen and its metabolites play a role in the development of PAH. High oestradiol (E2) and low dehydroepiandrosterone-sulfate (DHEA-S) levels have been identified as risk factors for PAH in males. More recently, high E2 and low DHEA-S levels have also been associated with the risk and severity of PAH in post-menopausal females [11]. It has been shown clinically that E2 is associated with PAH and correlates inversely with 6-min walk distance in males and post-menopausal females [12]. Additionally, anastrozole (ANA) improved the 6-min walk distance in a small-scale clinical trial of post-menopausal female and male PAH patients [13].

Our hypothesis was that obesity may induce changes in oestrogen metabolism, and that this could play a role in the development of pulmonary hypertension in obese males and females. To characterise the effects of obesity on endogenous oestrogen, and its contribution to PAH, the effects of an aromatase inhibitor, ANA, and the CYP1B1 inhibitor, 2,2′,4,6′-tetramethoxystilbene (TMS), were studied in obese mice. We focused on the leptin-deficient *ob*/*ob* mouse but also verified observations in mice fed a high-fat diet (HFD).

## Materials and methods

Detailed descriptions are provided in the supplementary material.

### *ob*/*ob* mice

B6.V-Lep*^ob^*/Lep*^ob^*/OlaHsd (*ob*/*ob*) mice aged 6–10 weeks and their lean littermates (B6.V-(lean)/OlaHsd) were obtained from Envigo (Huntingdon, UK).

### HFD mice

C57BL/6JOlaHsd mice aged 6–8 weeks old (Envigo) were maintained on a normal diet or a HFD (percentage calories from fat 42%, protein 15%, carbohydrate 43%; Special Diet Services, Witham, UK) for 20 weeks. The HFD mice gained 40% more body weight than their normal diet controls (supplementary figure S1).

### Chronic hypoxic studies

The development of pulmonary hypertension in *ob*/*ob* and HFD mice was achieved with 14 days hypoxia as described previously [14]. Mice were administered with the aromatase inhibitor ANA (3 mg·kg^−1^·day^−1^ for 14 days) or vehicle (1% carboxymethylcellulose) daily (intraperitoneally). Mice housed in normoxic conditions were studied as controls.

### Pharmacological inhibition of CYP1B1 in male *ob*/*ob* mice

Male *ob*/*ob* mice and their lean littermates were injected with TMS, a CYP1B1 inhibitor, 3 mg·kg^−1^·day^−1^, or vehicle (4% ethanol), once daily for 14 days (*i.p.*).

### Haemodynamic and right ventricular measurements

Right ventricular systolic pressure (RVSP) and systemic arterial pressure were measured using a PVR-1045 Millar pressure–conductance catheter (Millar Instruments, Houston, TX, USA). Right ventricular hypertrophy (RVH) was assessed as described previously [14].

### Lung histopathology

Lung sagittal sections (5 μm) were stained with Elastin–Van Gieson. Pulmonary arteries <80 μm external diameter were then microscopically assessed in a blinded fashion to assess vascular remodelling, as described in the supplementary material and previously [14].

### Measurement of E2, DHEA-S, testosterone and 16αOHE1

E2, DHEA-S, testosterone and 16αOHE1 levels were determined by ELISA as described in the supplementary material.

### Isolation and culture of mouse PASMCs

Mouse PASMCs (mPASMCs) were isolated from third-order pulmonary arteries of male lean and *ob*/*ob* mice, and used between passage 2 and 5.

### Preparation of visceral adipose tissue conditioned media

Cell culture media was incubated with visceral adipose tissue (VAT; 100 mg·mL^−1^ media) harvested from male *ob*/*ob* mice for 24 h at 37°C in the presence or absence of ANA or TMS. VAT conditioned media (VAT-CM) was diluted prior to use.

### Assessment of cell proliferation

Proliferation was assessed using a Countess II FL cell counter (Life Technologies, Loughborough, UK) as described in the supplementary material.

### Immunoblotting

Proteins of interest were assessed by immunoblotting whole lung or mPASMC lysates as described in the supplementary material.

### Quantitative reverse transcriptase-PCR

mRNA expression was assessed by quantitative reverse transcriptase-PCR as described in the supplementary material.

### Amplex Red assay

Hydrogen peroxide (H_2_O_2_) was assessed in mPASMC lysates using an Amplex Red Hydrogen Peroxide/Peroxidase Assay Kit (Thermo Fischer Scientific, Loughborough, UK) according to the manufacturer's instructions.

### Protein tyrosine phosphatase oxidation assessment

Briefly, irreversible oxidation of protein tyrosine phosphatases (PTPs) was assessed by immunoblotting using an oxidised PTP antibody that specifically recognises the sulfonic acid form of PTP cysteine residues.

### ROS determination in lung sections by immunofluorescence

Immunohistochemistry of the ROS marker 8-hydroxyguanosine (8-OHG) was determined in whole lung sections as described previously [10].

### Data analysis

All data are expressed as mean with standard error of the mean. Data were analysed using one-way or two-way ANOVA with *post hoc* analyses or the unpaired t-test (as appropriate and indicated in the figure legends) using Prism version 5 (GraphPad, La Jolla, CA, USA). A p-value <0.05 was considered statistically significant.

## Results

### Aromatase expression is upregulated in VAT of male obese mice

**FIGURE 1:**
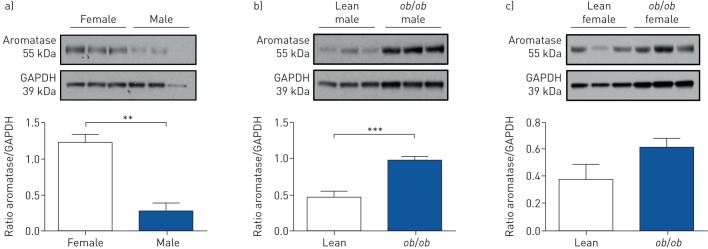
Characterisation of changes in aromatase expression in visceral adipose tissue (VAT) from lean *versus* obese mice. GAPDH: glyceraldehyde phosphate dehydrogenase. Representative immunoblot and quantification of aromatase protein expression in VAT from a) lean female and male mice, b) lean and *ob*/*ob* male mice, and c) lean and *ob*/*ob* female mice (n=3–4 per group). Data are presented as mean±sem. **: p<0.01; ***: p<0.001, determined by two-tailed unpaired t-test.

Peri-renal adipose tissue is an example of white VAT, and allows direct comparison between males and females. Lean female mice express greater levels of aromatase in VAT than males (figure 1a). Aromatase expression was elevated in VAT from male *ob*/*ob* but not female *ob*/*ob* mice compared with their lean controls (figure 1b and c). Increased aromatase expression was also confirmed in VAT from male HFD mice, but not females (supplementary figure S2).

### Inhibition of aromatase attenuates parameters of pulmonary hypertension in both male and female obese mice

**FIGURE 2:**
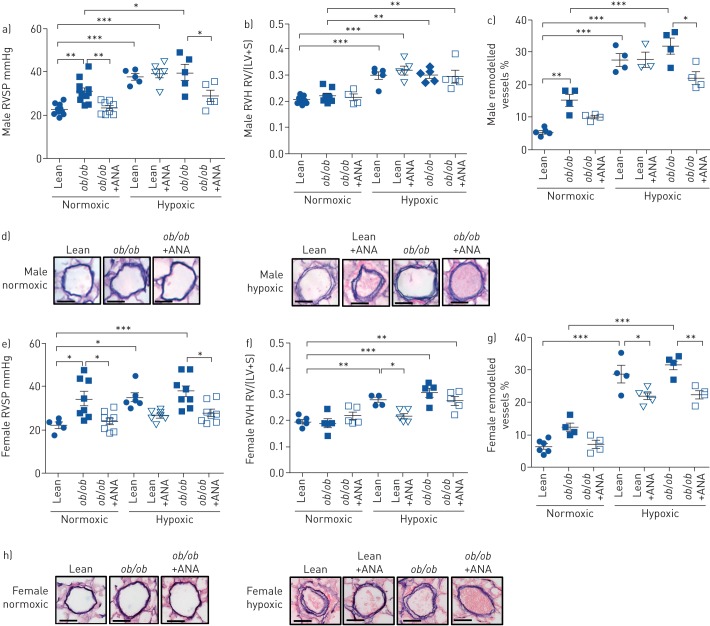
Inhibition of aromatase attenuates parameters of pulmonary hypertension in *ob*/*ob* mice. RVSP: right ventricular systolic pressure; ANA: anastrozole; RVH: right ventricular hypertrophy; RV/(LV+S): right ventricle/(left ventricle+septum) ratio. a–d) Male: effects of ANA 3 mg·kg^−1^·day^−1^ for 14 days on a) RVSP (n=5–10 per group), b) RVH (n=5–10 per group) (as determined by RV/(LV+S) ratio) and c) percentage of remodelled pulmonary arteries (n=4 per group) in normoxic and hypoxic male *ob*/*ob* mice. d) Representative images of pulmonary arteries from normoxic and hypoxic male *ob*/*ob* mice treated with or without ANA 3 mg·kg^−1^·day^−1^. Scale bar: 50 µm. e–h) Female: effects of ANA 3 mg·kg^−1^·day^−1^ for 14 days on e) RVSP (n=5–10 per group), f) RVH (n=5–10 per group) (as determined by RV/(LV+S) ratio) and g) percentage of remodelled pulmonary arteries (n=4 per group) in normoxic and hypoxic female *ob*/*ob* mice. h) Representative images of pulmonary arteries from normoxic and hypoxic female *ob*/*ob* mice treated with or without ANA 3 mg·kg^−1^·day^−1^. Scale bar: 50 µm. Data are presented as mean±sem. *: p<0.05; **: p<0.01; ***: p<0.001, determined by one-way ANOVA with Bonferroni post-test.

An increase in RVSP was observed in male *ob*/*ob* mice under normoxic conditions, whereas RVH remained unchanged (figure 2a and b). This increase in RVSP was reversed by ANA (figure 2a). An increase in pulmonary vascular remodelling was also observed in normoxic male *ob*/*ob* mice and this was unaffected by ANA treatment (figure 2c and d). ANA had no effect on hypoxia-induced changes in RVSP, RVH and pulmonary vascular remodelling in lean male mice, but attenuated hypoxia-induced increases in RVSP and vascular remodelling in male *ob*/*ob* mice (figure 2a–d).

Similar findings were observed in normoxic female *ob*/*ob* mice compared with males. However, in contrast to males, ANA decreased RVH and pulmonary vascular remodelling in female lean mice, and was effective in attenuating RVSP, RVH and pulmonary vascular remodelling in female *ob*/*ob* mice (figure 2e–h).

No significant changes in mean systemic arterial pressure, left ventricular end-diastolic pressure (LVEDP) or left ventricle plus septum (LV+S) weight were observed between groups in males or females (supplementary figure S3).

To further assess right ventricular function, gene expression analysis of markers of heart failure and fibrosis was carried out in vehicle- and ANA-treated *ob*/*ob* mice. Fibronectin, connective tissue growth factor, atrial natriuretic peptide and brain natriuretic peptide expression levels were assessed. Changes in right ventricular remodelling were also determined by Picrosirius Red staining. No significant changes in right ventricular remodelling or gene expression were observed (supplementary figure S4).

Following 20 weeks on a HFD, male mice displayed no significant increases in RVSP or pulmonary vascular remodelling, although an increase in RVH was observed (supplementary figure S5a–d). Under hypoxic conditions, male HFD mice developed significantly more pulmonary vascular remodelling than those on a normal diet (supplementary figure S5d). Administration of ANA attenuated hypoxia-induced increases in RVSP, RVH and pulmonary vascular remodelling in male HFD mice (supplementary figure S5a–d). Similarly, female mice showed no significant changes in RVSP or pulmonary vascular remodelling following a HFD; however, RVH was significantly increased. ANA reduced RVSP in females (supplementary figure S5e–h). No differences in mean systemic arterial pressure, LV+S weight or LVEDP (supplementary figure S6) were observed between groups in males or females.

Both *ob*/*ob* and HFD mice displayed increased body weight compared with their lean controls. ANA treatment decreased body weight in hypoxic male *ob*/*ob* mice and female normoxic *ob*/*ob* mice only (supplementary figure S7).

### Effects of obesity on circulating E2 and testosterone levels

**FIGURE 3:**
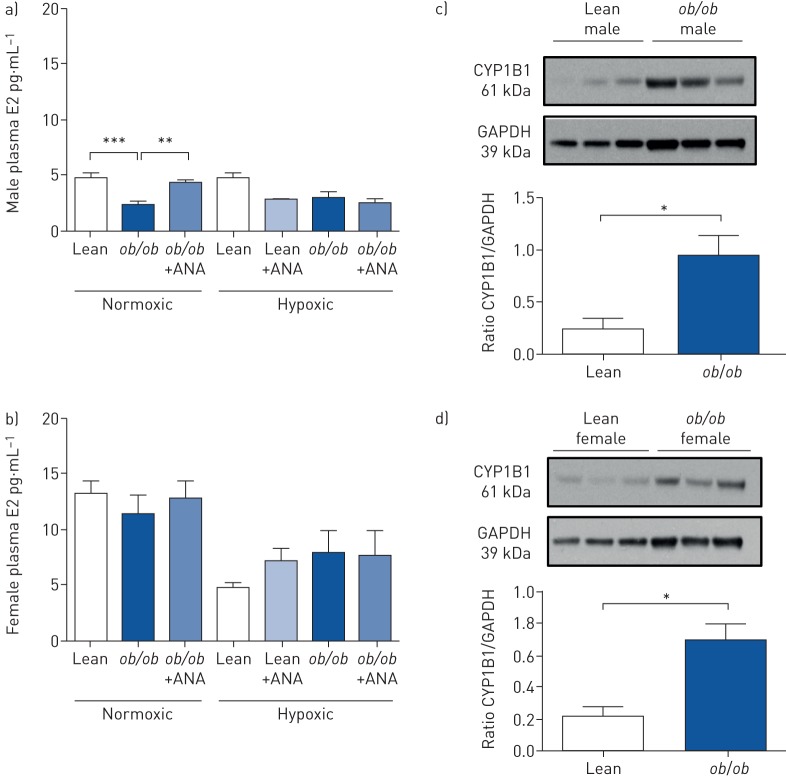
Effects of obesity on circulating oestradiol (E2) and visceral adipose tissue (VAT) expression of cytochrome P450 1B1 (CYP1B1). ANA: anastrozole; GAPDH: glyceraldehyde phosphate dehydrogenase. a, b) Circulating plasma E2 levels in a) normoxic and hypoxic lean and *ob*/*ob* male mice (n=5–10 per group) and b) normoxic and hypoxic lean and *ob*/*ob* female mice (n=4–7 per group) treated with or without ANA 3 mg·kg^−1^·day^−1^. c, d) Representative immunoblot and quantification of CYP1B1 protein expression in VAT from c) male and d) female lean and *ob*/*ob* mice (n=3–4 per group). Data are presented as mean±sem. *: p<0.05; **: p<0.01; ***: p<0.001, determined by one-way ANOVA with Bonferroni post-test.

A decrease in plasma E2 was detected in normoxic *ob*/*ob* male mice compared with their lean controls (figure 3a). Hypoxia alone, or in the presence of ANA, had no effect on circulating E2 levels in either lean or *ob*/*ob* mice (figure 3a).

In *ob*/*ob* females, no changes in plasma E2 were observed between the groups (figure 3b). Similarly, plasma E2 was decreased in HFD males compared with their lean controls and E2 levels were unaffected by hypoxia alone or ANA treatment (supplementary figure S8a). Neither a HFD nor hypoxia had any effect on plasma E2 levels in females. However, in female HFD hypoxic mice, ANA treatment reduced the levels of circulating E2 (supplementary figure S8b).

Uterus weights in lean mice and their E2 levels suggest they are in oestrus, and the E2 concentrations observed are comparable with those previously detected in female mice (∼0.3–10 pg·mL^−1^) [15, 16]. Additionally, *ob*/*ob* females are infertile and have significantly lower uterine weights (supplementary figure S9a). Uterine weight was unaffected by a HFD, but reduced by ANA (supplementary figure S9b).

Testosterone levels were also assessed in male *ob*/*ob* and HFD mice. No significant differences in testosterone were observed between *ob*/*ob* and HFD study groups (supplementary figure S9c and d).

We were unable to detect DHEA-S in plasma samples from the mice studied (data not shown).

### CYP1B1 and 16αOHE1 are increased in obese male mice

**FIGURE 4:**
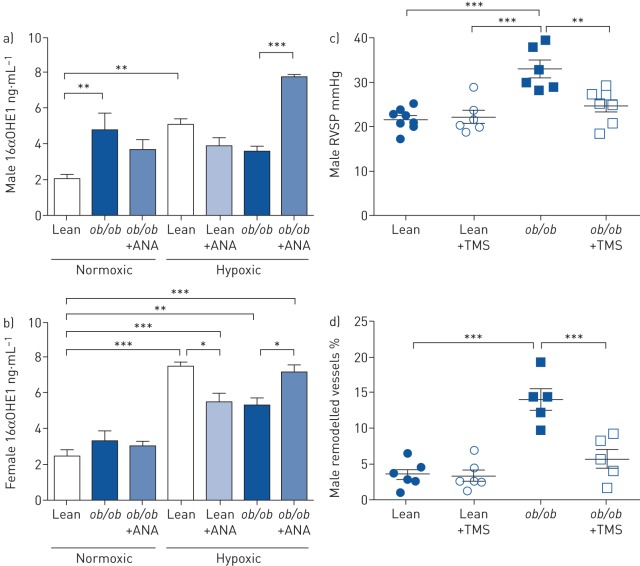
16α-hydroxyestrone (16αOHE1) levels and the effects of cytochrome P450 1B1 (CYP1B1) inhibition in obese mice. ANA: anastrozole; RVSP: right ventricular systolic pressure. a, b) Urinary 16αOHE1 levels in a) male and b) female *ob*/*ob* mice treated with and without ANA 3 mg·kg^−1^·day^−1^ for 14 days in normoxic and hypoxic conditions (n=4–8 per group). c, d) The effects of CYP1B1 inhibition with TMS 3 mg·kg^−1^·day^−1^ for 14 days on c) RVSP (n=5–7 per group) and d) percentage of remodelled pulmonary arteries (n=5 per group) in male normoxic lean and *ob*/*ob* mice. Data are presented as mean±sem. *: p<0.05; **: p<0.01; ***: p<0.001, determined by one-way ANOVA with Bonferroni post-test.

CYP1B1 expression was elevated in VAT from male and female *ob*/*ob* mice compared with their lean controls (figure 3c and d). In keeping with this observation, urinary 16αOHE1 levels were elevated in male normoxic *ob*/*ob* mice (figure 4a). In the male study groups, hypoxia resulted in an increase in urinary 16αOHE1 in lean animals that was unaffected by ANA treatment. However, ANA did increase urinary 16αOHE1 in hypoxic *ob*/*ob* males (figure 4a). No changes in 16αOHE1 were observed in female *ob*/*ob* animals under normoxic conditions. In the female hypoxic study groups, hypoxia resulted in an increase in 16αOHE1 and this was attenuated by ANA treatment in lean mice, whilst ANA augmented 16αOHE1 in *ob*/*ob* mice (figure 4b).

CYP1B1 expression was increased in VAT from HFD males; however, no comparable increase was apparent in HFD female mice (supplementary figure S10a and b). Similar to normoxic *ob*/*ob* males, a HFD resulted in an increase in urinary 16αOHE1 levels. In the case of the HFD study, only lean male animals displayed an increase in 16αOHE1 levels in hypoxia. This hypoxia-induced increase in 16αOHE1 was attenuated in male HFD animals and ANA treatment had no effect on this (supplementary figure S10c). No effect of hypoxia or ANA treatment on 16αOHE1 was observed in the female arm of the HFD study (supplementary figure S10d).

16αOHE1 is often reported as the ratio of 2OHE1 to 16αOHE1. However, the levels of 2OHE1 are often very low in mouse plasma and we are not able to consistently detect its presence by the ELISA method used. Therefore, results are expressed as 16αOHE1 concentrations only.

### Pharmacological inhibition of CYP1B1 attenuates the pulmonary hypertension phenotype in male *ob*/*ob* mice

Given the significant increase in CYP1B1 and its product 16αOHE1 in obese male mice we assessed the effect of TMS, a selective CYP1B1 inhibitor, on the pulmonary hypertension phenotype observed in male *ob*/*ob* mice. TMS treatment significantly attenuated the elevated RVSP and pulmonary vascular remodelling observed in male *ob*/*ob* mice (figure 4c and d).

Large amounts of intra-thoracic fat were observed in the *ob*/*ob* mice studied, but this was not so apparent in lean animals. Intra-thoracic fat from male *ob*/*ob* mice was found to contain 0.94±0.19 ng·mL^−1^ 16αOHE1 (n=5).

### Effects of VAT-CM on mPASMC proliferation and oxidative stress

**FIGURE 5:**
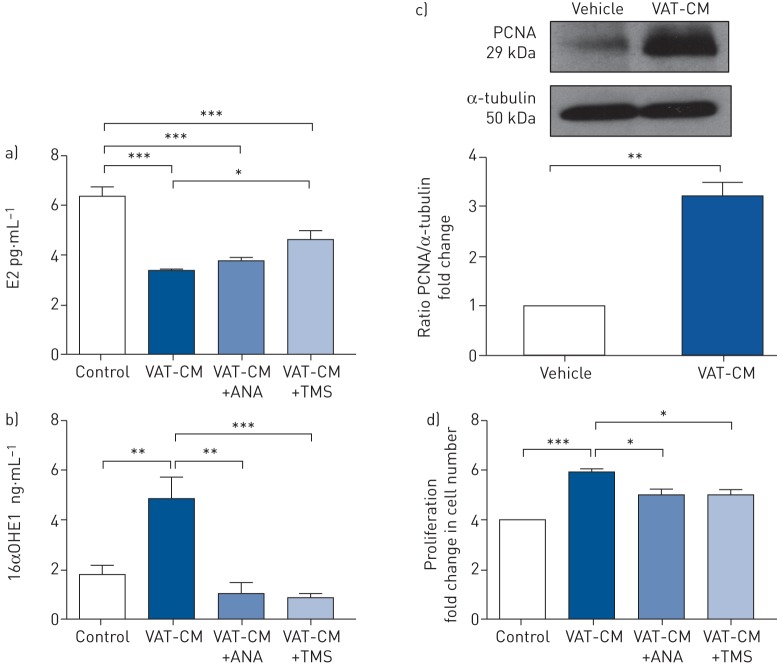
Effects of visceral adipose tissue conditioned media (VAT-CM) on mouse pulmonary artery smooth muscle cell (mPASMC) proliferation. E2: oestradiol; ANA: anastrozole; TMS: 2,2′,4,6′-tetramethoxystilbene; 16αOHE1: 16α-hydroxyestrone; PCNA: proliferating cell nuclear antigen. a) E2 levels and b) 16αOHE1 levels in control media and VAT-CM prepared in the absence or presence of ANA or TMS (n=4 per group). c) Representative immunoblot and quantification of PCNA expression in mPASMCs following 24 h treatment with VAT-CM (n=3 per group). d) The effects of 24 h VAT-CM prepared in the presence or absence of ANA or TMS on mPASMC proliferation (data normalised to control group, n=3 per group). Data are presented as mean±sem. *: p<0.05; **: p<0.01; ***: p<0.001, determined by two-tailed unpaired t-test or one-way ANOVA with Bonferroni post-test, as appropriate.

To investigate the role of CYP1B1 in male *ob*/*ob* mice further, cell culture media was incubated with VAT harvested from male *ob*/*ob* mice. Analysis of the VAT-CM revealed it contained significantly lower levels of E2 compared with control media (figure 5a). ANA pre-treatment had no effect on this, but TMS resulted in a significant increase in E2 levels (figure 5a). Conversely, VAT-CM contained significantly higher levels of 16αOHE1 compared with control media, and this was attenuated by both ANA and TMS (figure 5b). The stimulation of mPASMCs with VAT-CM increased proliferating cell nuclear antigen (PCNA) expression (figure 5c) and resulted in cell proliferation. The proliferative effects of VAT-CM prepared in the presence of ANA or TMS were significantly reduced (figure 5d).

**FIGURE 6:**
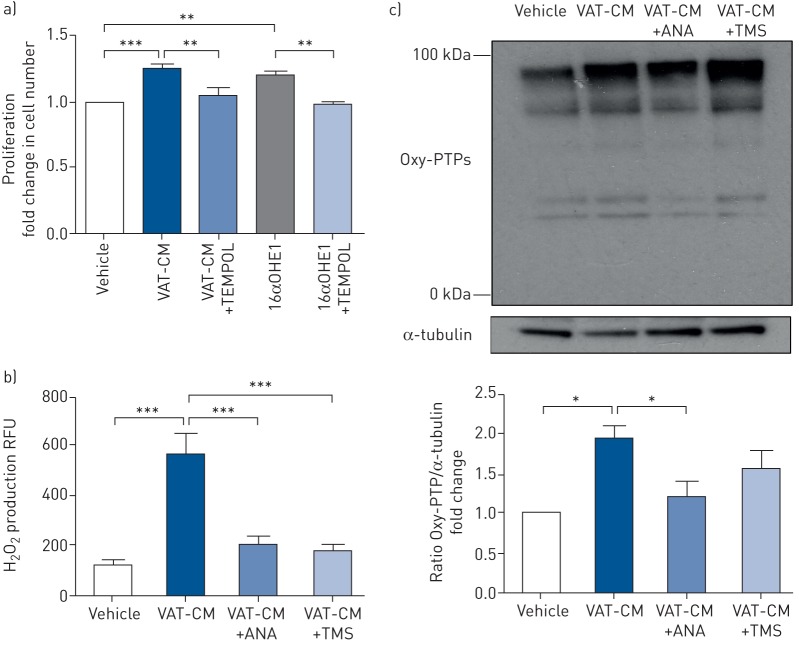
Effects of visceral adipose tissue conditioned media (VAT-CM) stimulation on markers of oxidative stress. TEMPOL: 4-hydroxy-TEMPO; 16αOHE1: 16α-hydroxyestrone; H_2_O_2_: hydrogen peroxide; RFU: relative fluorescence unit; ANA: anastrozole; TMS: 2,2′,4,6′-tetramethoxystilbene; Oxy-PTP: oxidised protein tyrosine phosphatase; mPASMC: mouse pulmonary artery smooth muscle cell. a) The effects of 24 h VAT-CM and 16αOHE1 (1 nM) stimulation in the presence or absence of TEMPOL (10 µM) on mPASMC proliferation (data normalised to vehicle group, n=5 per group). b, c) The effect of 24 h stimulation with VAT-CM prepared in the presence of absence of ANA or TMS on b) H_2_O_2_ production (expressed as RFUs) and c) Oxy-PTPs in mPASMCs (Oxy-PTP blot shown is representative of n=3 experiments as quantified in the corresponding histogram with data normalised to vehicle group). All data are presented as mean±sem. *: p<0.05; **: p<0.01; ***: p<0.001, determined by one-way ANOVA with Bonferroni or Dunnett *post hoc* analyses.

**FIGURE 7:**
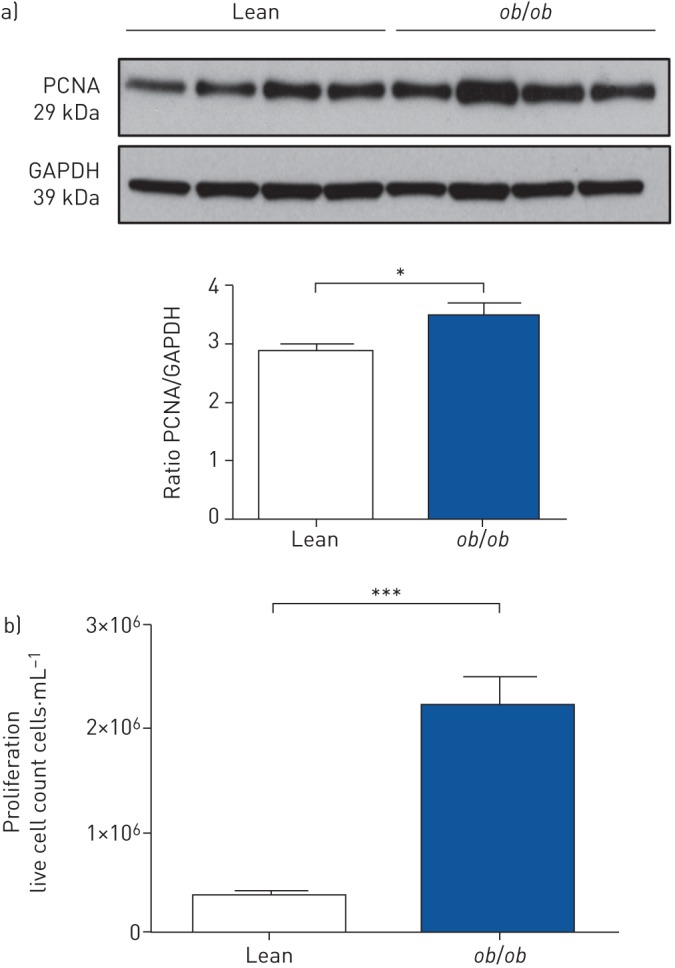
Mouse pulmonary artery smooth muscle cells (mPASMCs) isolated from male *ob*/*ob* mice are more proliferative than mPASMCs from their lean littermates. PCNA: proliferating cell nuclear antigen; GAPDH: glyceraldehyde phosphate dehydrogenase. a) Representative immunoblot and quantification (n=4) of PCNA expression, and b) proliferation (expressed as live cell count in cells·mL^−1^) of lean *versus ob*/*ob* male mPASMCs after 48 h in normal growth conditions. Data are presented as mean±sem. *: p<0.05; ***: p<0.001, determined by two-tailed unpaired t-test.

VAT-CM induced proliferation to a similar extent as 16αOHE1 and this was attenuated by the ROS scavenger, 4-hydroxy-TEMPO (figure 6a). Furthermore, VAT-CM induced H_2_O_2_ production and irreversible oxidative modification of PTPs, a marker of oxidative stress (figure 6b and c). mPASMCs isolated from male *ob*/*ob* mice displayed increased levels of PCNA and were more proliferative than mPASMCs from lean mice (figure 7a and b).

### Effects of ANA on obesity-induced oxidative damage in mouse lung

**FIGURE 8:**
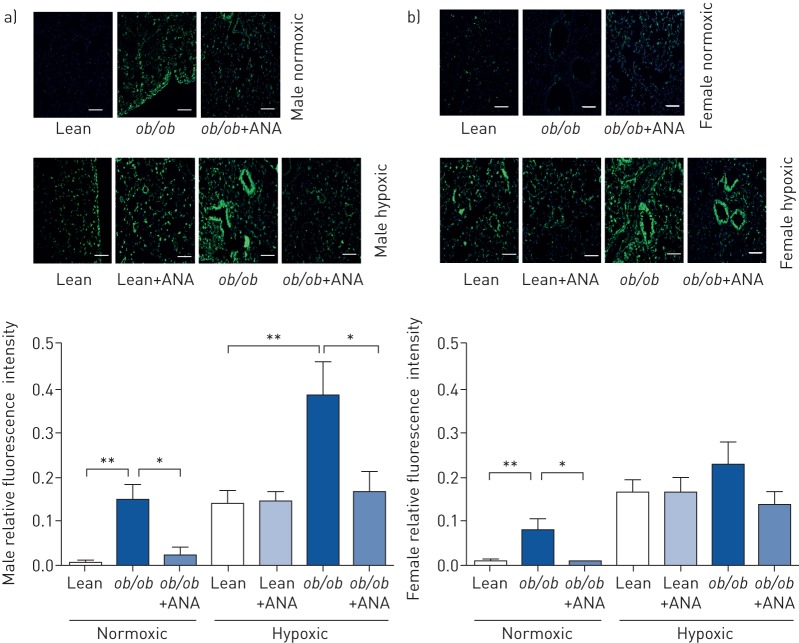
Effects of aromatase inhibition on obesity-induced oxidative damage in mouse lung. ANA: anastrozole; 8-OHG: 8-hydroxyguanosine. Representative images of reactive oxygen species marker 8-OHG and corresponding quantification in whole lung sections from a) male and b) female lean and *ob*/*ob* mice treated with or without ANA. Green: 8-OHG; blue: 4′,6-diamidino-2-phenylindole. Scale bar: 50 µm. *: p<0.05; **: p<0.01, determined by one-way ANOVA with Bonferroni post-test of normoxic and hypoxic groups independently.

Immunofluorescence of the ROS marker 8-OHG was determined in whole lung sections of male *ob*/*ob* mice. An increase in 8-OHG immunofluorescence was observed in normoxic male *ob*/*ob* mice and this was attenuated by ANA (figure 8a). Hypoxia-induced increases in 8-OHG staining were unaffected by ANA in lean mice. In hypoxic male *ob*/*ob* mice, 8-OHG staining was increased compared with lean hypoxic males and this was attenuated by ANA treatment (figure 8a). Similar results were observed in normoxic *ob*/*ob* females, but no differences in hypoxic female mice with or without ANA were determined (figure 8b).

Changes in antioxidant enzymes were also assessed in the lungs from *ob*/*ob* mice and no significant differences in superoxide dismutase 1 (SOD1) or catalase were observed between the groups in male animals (supplementary figure S11a and b). NADPH oxidase 4, which generates intracellular superoxide, was significantly increased in the lung tissue of male hypoxic *ob*/*ob* mice compared with normoxic and this was attenuated by ANA (supplementary figure S11c). In female lung, ANA treatment resulted in increases in SOD1 in normoxic conditions and catalase expression in hypoxic conditions (supplementary figure S11d and e). Antioxidant enzymes were also assessed in VAT; SOD1 was found to be decreased in *ob*/*ob* mice and this was unchanged by ANA (supplementary figure S12a). Catalase and glutathione peroxidase expression levels were unchanged in *ob*/*ob* VAT and unaffected by ANA (supplementary figure S12b and c)

## Discussion

Adipose tissue is metabolically active, expressing high levels of aromatase within its stromal fraction, and is a known source of oestrogen production [4]. Given that 32% of PAH patients may be obese, as reported by the REVEAL registry [1], the current pre-clinical study was designed to assess the potential effect of obesity on changes in oestrogen metabolism and the pathogenesis of experimental pulmonary hypertension in males and females.

**FIGURE 9:**
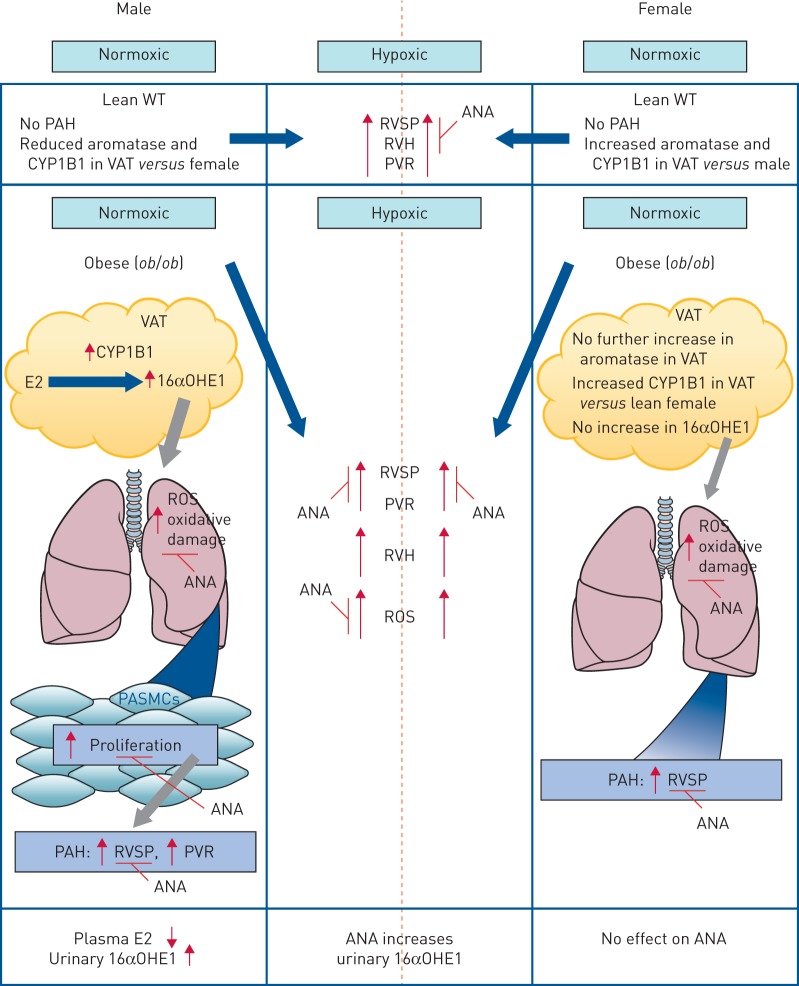
Summary of results in wild-type (WT) lean and *ob*/*ob* mice. PAH: pulmonary arterial hypertension; CYP1B1: cytochrome P450 1B1; VAT: visceral adipose tissue; RVSP: right ventricular systolic pressure; RVH: right ventricular hypertrophy; PVR: pulmonary vascular remodelling; ANA: anastrozole; E2: oestradiol; 16αOHE1: 16α-hydroxyestrone; ROS: reactive oxygen species; PASMC: pulmonary arterial smooth muscle cell. Where inhibition by ANA is indicated this suggests dependency on endogenous oestrogen (E2). In normoxic female *ob*/*ob* mice there is E2-dependent increased RVSP. Aromatase expression in VAT is increased in WT lean females compared with males and does not increase further with obesity. Plasma levels of E2 are higher in lean WT females than males and unaffected by obesity. In hypoxia there are E2-dependent increases in RVSP and PVR. There is also E2-dependent ROS-driven oxidative damage *in vivo*. *ob*/*ob* male mice demonstrate E2-dependent elevations in RVSP and this is accompanied by increased aromatase expression in adipose tissue. There is, however, a decrease in circulating E2. CYP1B1 expression is increased in VAT and consistent with this there is an increase in urinary 16αOHE1. In hypoxia there are also E2-dependent increases in RVSP and PVR. VAT from male *ob*/*ob* mice converts E2 to 16αOHE1 *via* CYP1B1. This 16αOHE1 is produced in sufficient amounts to cause CYP1B1-dependent proliferation of PASMCs. This proliferation was greatest in *ob*/*ob* mice and was ROS dependent. *In vivo*, ROS lung production in male *ob*/*ob* mice was dependent on endogenous E2, as was hypoxia-induced ROS.

Our results provide novel mechanistic insight into epidemiological observation linking pulmonary hypertension and obesity. As we studied many variables *in vivo*, the results are complex. In summary, however, the main findings were that, in normoxic female *ob*/*ob* mice, there is E2-dependent increased RVSP. Aromatase expression in VAT is 4–5-fold higher in lean females than males and does not increase further with obesity. Plasma levels of E2 are some 3-fold higher in females than males and unaffected by obesity. Although VAT CYP1B1 is elevated this did not result in any depletion of E2 plasma levels or changes in urinary 16αOHE1. This suggests that CYP1B1 activity may not drive the obesity-induced changes in RVSP in females. This is summarised in figure 9. In hypoxia there are also E2-dependent increases in RVSP and vascular remodelling.

*ob*/*ob* male mice demonstrate E2-dependent elevations in RVSP and this is accompanied by increased aromatase expression in adipose tissue. There is, however, a decrease in circulating E2. As there is increased CYP1B1 expression in VAT this suggests that there may be increased metabolism of E2 and consistent with this there is an increase in urinary 16αOHE1. Inhibition of CYP1B1 with TMS also reversed the obesity-induced increase in RVSP in males. This is summarised in figure 9. In hypoxia there are also E2-dependent increases in RVSP and vascular remodelling.

As obesity in male mice may be uniquely driven by CYP1B1-induced E2 metabolism we focused on the *ob*/*ob* males to investigate this further. In summary, we demonstrated that VAT from male *ob*/*ob* mice converts E2 to 16αOHE1 *via* CYP1B1. This 16αOHE1 is produced in sufficient amounts to cause CYP1B1-dependent proliferation of PASMCs. This proliferation was greatest in *ob*/*ob* mice and was ROS dependent. *In vivo*, ROS lung production in male *ob*/*ob* mice was dependent on endogenous E2, as was hypoxia-induced ROS. This is summarised in figure 9.

Both male and female *ob*/*ob* mice develop pulmonary hypertension spontaneously, an effect that can be attenuated by aromatase inhibition, suggesting a role for endogenous oestrogen in both male and female obese mice. This is consistent with the observation that in humans, increased body mass index is associated with an increase in pulmonary arterial systolic pressure in both males and females [17]. We have previously demonstrated that ANA treatment is only therapeutic in female hypoxic rodents and not males [18]. This is due to the unique phenotype of female PASMCs, whereby endogenous oestrogen produced by aromatase in these cells predisposes female PASMCs to proliferation and the development of pulmonary hypertension [18, 19]. Thus, the ability of ANA to attenuate pulmonary hypertension in obese hypoxic male mice but not lean as observed in the current study suggests that endogenous oestrogens are involved in the development of pulmonary hypertension in obese males. This may be due to obesity-mediated changes in oestrogen metabolism. In males, ∼60% of circulating oestradiol is derived from direct testicular secretion or from conversion of testicular androgens and the remainder derived from peripheral conversion of adrenal androgens [20]. It has been suggested that aromatase is less suppressed in the testis compared with adipose and muscle tissue by third-generation aromatase inhibitors such as ANA [21]. Therefore, the increased peripheral production of oestrogen and it metabolites in obese mice may account for the therapeutic effect of aromatase inhibition observed in obese but not lean mice in this study.

Previous studies using *ob*/*ob* mice have yielded conflicting results regarding the development of pulmonary hypertension. The *ob*/*ob* genotype has been reported to attenuate hypoxia-induced pulmonary hypertension by inhibiting proliferation of PASMCs [22], while another study suggests it recapitulates many of the histological features of pulmonary hypertension [23]. We therefore chose to further investigate the effects of obesity on pulmonary hypertension using a diet-induced model of obesity where mice were maintained on a HFD. As previously reported, a HFD on its own did not induce pulmonary hypertension [24]. Therefore, we studied the effect of a HFD on the development of pulmonary hypertension in hypoxic conditions. In this instance, obese males and females developed significantly more pulmonary vascular remodelling than lean males. ANA attenuated parameters of pulmonary hypertension in both males and females, but appeared to be more beneficial in males, attenuating all pulmonary hypertension indices. In females, ANA only decreased RVSP, and had no effect on RVH and pulmonary vascular remodelling. We also confirmed that in male HFD obese mice there was a decrease in plasma E2 with an increase in urinary 16αOHE1, and that VAT expressed increased aromatase and CYP1B1 (supplementary figure S13). These findings support the hypothesis of sexual dimorphism in the mechanisms underlying pulmonary hypertension that may be accentuated in obesity due to the pronounced changes in oestrogen metabolism observed in males.

The authors acknowledge that the phenotype observed in the mouse models used in this study is not as severe as that seen in other hypoxic models. However, it is comparable to other transgenic mouse models that have not been exposed to hypoxia, such as SERT^+^ mice [6], Smad-1^+/−^ mice [19] and bone morphogenetic protein receptor type 2 (BMPR2) R899X mice [25]. The pulmonary hypertension phenotype observed is not due to left-sided heart failure as no changes in LV+S weight, LVEDP or systemic arterial pressure were detected between lean and *ob*/*ob* mice. Indeed, it has been previously documented that *ob*/*ob* mice maintain normal systemic blood pressure despite displaying severe obesity [26]. Additionally, no significant differences in markers of right ventricular remodelling were observed between sexes or ANA treatment groups. Furthermore, the ability of ANA to attenuate pulmonary hypertension in obese male mice but not lean suggests that inhibition of aromatase may influence obesity-induced elevations in pulmonary arterial pressures and thereby modify the mechanisms underlying pulmonary hypertension development. ANA treatment resulted in a reduction in body weight in normoxic *ob*/*ob* females and hypoxic *ob*/*ob* males that may also have contributed to the decrease in RVSP in these groups given the association of body mass index with pulmonary arterial systolic pressure.

The disparity between increased levels of aromatase in VAT and the decrease in circulating E2 levels suggests oestrogen may be metabolised in VAT rather than excreted. Therefore, we assessed the expression of the oestrogen-metabolising enzyme CYP1B1 in VAT from the obese models studied. CYP1B1 has previously been reported to be highly expressed in VAT and its expression increases during adipogenesis [27]. Here, we demonstrate that CYP1B1 is upregulated in VAT from obese *ob*/*ob* and HFD male mice, and that this correlates with an increase in urinary 16αOHE1 levels. Low DHEA-S has been identified as a risk factor for PAH in male patients; however, the circulating levels of DHEA-S in the mice studied were below the limits of detection of the assay used. Others have also reported low or undetectable DHEA-S in mice [15]. Changes in testosterone were also assessed and no significant difference observed between the treatment groups of mice studied. This suggests changes specific to oestrogen and its metabolism by CYP1B1, rather than their upstream mediators, occur in the obese models studied. The pathogenic role of CYP1B1 and 16αOHE1 in obese mice was confirmed by the beneficial effect of the CYP1B1 inhibitor TMS observed in this study. Changes in oestrogen metabolism have previously been observed in PAH. CYP1B1 is highly upregulated within pulmonary arterial lesions of PAH patients and pharmacological inhibition of CYP1B1 can attenuate the development of experimental pulmonary hypertension. These studies have also demonstrated increased pulmonary arterial expression of CYP1B1 in lungs from hypoxic mice (males and females) and the Sugen/hypoxic mouse model (males and females) [8, 28]. Oestrogen metabolism is a strong predictor of penetrance in heritable PAH. Polymorphisms in CYP1B1 that cause preferential metabolism of oestrogen to 16αOHE1 result in the development of PAH in females, whereas females who preferentially metabolise oestrogen into 2OHE1 or 4OHE1 do not [29, 30]. Oestrogen metabolism can drive PAH penetrance in males, but not to the same degree as in females [31].

The increased CYP1B1 expression in VAT and the subsequent increase in urinary 16αOHE1 levels observed in male *ob*/*ob* and HFD mice may directly contribute to the pulmonary hypertension phenotype observed in these animals, given the beneficial effects of CYP1B1 inhibition on pulmonary hypertension observed in *ob*/*ob* mice. Intra-thoracic fat present in male *ob*/*ob* mice contains 16αOHE1. As this VAT is in direct contact with both heart and lung tissue it may have significant effects on these tissues and contribute to the development of pulmonary hypertension. Further contributing to this hypothesis is the finding that VAT-CM has significantly lower levels of E2 and higher levels of 16αOHE1 than control media, an effect that can be attenuated by CYP1B1 inhibition. This suggests VAT can metabolise E2 *via* CYP1B1, resulting in the secretion of 16αOHE1. ANA was effective at reducing indicators of pulmonary hypertension in the hypoxic male *ob*/*ob* and hypoxic HFD male mice, whereas it was not effective in the male lean hypoxic mice. This suggests that in hypoxia, endogenous oestrogen plays an increased role in the development of pulmonary hypertension in male obese mice. The relationships between the development of pulmonary hypertension, CYP1B1 and effectiveness of ANA are, however, less clear in hypoxic male mice. Hypoxia itself increased urinary 16αOHE1 in the lean males, but in the *ob*/*ob* male mice this was not increased any further. A HFD in male mice actually reduced the elevated urinary 16αOHE1 seen in hypoxia. This suggests that hypoxia itself affects oestrogen metabolism and that endogenous oestrogen is exerting pathogenic effects independent of CYP1B1 activity in hypoxic obese males. Hypoxia and oestrogen are known to reduce BMPR2 signalling [32, 33]. Indeed, BMPR2 signalling is reduced in male and female hypoxic mouse lung [18]. In male hypoxic mice, hypoxia elevates 16αOHE1, which has been shown to synergise with BMPR2 deficiency and uncover a pulmonary hypertension phenotype in BMPR2-deficient transgenic mice. This pulmonary hypertension phenotype in BMPR2-deficient mice is reversed by ANA [34]. We know that oestrogen synthesis occurs *via* aromatase in the hypoxic mouse lung [18]. Hence, endogenous oestrogen and increased 16αOHE1 may be synergising with reduced BMPR2 in obese hypoxic mice to contribute to the pulmonary hypertension phenotype. Paradoxically, ANA increased urinary 16αOHE1 in both male and female mice while maintaining levels of plasma oestrogen. This suggests that the effectiveness of ANA at reversing pulmonary hypertension in these hypoxic mice is closely related to the effects of hypoxia on lung oestrogen synthesis *via* aromatase and the direct effects of this endogenous oestrogen on lung pathology and BMPR2 signalling. These effects may be more influential than the effects of dysregulated oestrogen metabolism.

The results do suggest, however, a close relationship between adipose tissue CYP1B1 expression, the development of pulmonary hypertension and effectiveness of ANA in normoxic *ob*/*ob* male mice. Indeed, incubation of PASMCs with VAT-CM leads to ROS generation, oxidative damage and activation of cell survival pathways, resulting in their proliferation in a redox-sensitive manner similar to that observed with 16αOHE1 alone. These effects are attenuated by both aromatase and CYP1B1 inhibition. In keeping with this finding, an increase in 8-OHG staining, a marker of ROS production, was observed in whole lung tissue from *ob*/*ob* mice. ANA treatment significantly attenuated this phenomenon in male and female mice in normoxia, but was only effective in male hypoxic mice, again highlighting the sexual dimorphism in mechanisms underpinning pulmonary hypertension development. No significant changes in the antioxidant enzymes SOD1 or catalase were observed in lung tissue of the mice studied; however, NADPH oxidase 4 was upregulated in male *ob*/*ob* mice in hypoxia and this was attenuated by ANA. These findings suggest an increase in ROS production rather than a decrease in the levels of antioxidants is occurring in obesity and that ANA has antioxidant properties.

We have previously comprehensively demonstrated mechanisms of 16αOHE1-induced proliferation and redox signalling in PASMCs, and so have not addressed this in the current study. 16αOHE1 increases NADPH oxidase 1 expression and ROS production, leading to irreversible oxidation of PTPs, decreased activity nuclear factor erythroid-related factor 2 (Nrf2) and increased cell proliferation in human PASMCs [10]. The signalling pathways stimulated by 16αOHE1 are unique to the pulmonary circulation as 16αOHE1 failed to induce ROS production or proliferation in vascular smooth muscle cells from the systemic circulation [10]. Although the effects of 16αOHE1 on ROS production appear to be specific to the pulmonary circulation, increases in ROS production have been observed globally in obesity [35]. Indeed, levels of the antioxidant enzyme SOD1 are reduced in VAT from male *ob*/*ob* mice. ROS production is also known to be increased in hypoxia in a variety of tissues [36], and this may well have systemic effects in the obese models studies and drive other changes in oestrogen responsiveness and metabolism that account for some of the differences in 16αOHE1 observed in hypoxic animals. The effects of hypoxia on oestrogen metabolism have been most widely studied in cancer cells, where it is known to alter responsiveness to oestrogen and its metabolism [37]. The beneficial effects of ANA observed may therefore be due to a general reduction in ROS as well as a reduction in 16αOHE1-mediated ROS production.

16αOHE1 is also known to promote insulin resistance and other metabolic disorders, and the *ob*/*ob* mice used in this study are indeed glucose intolerant. Therefore, it is possible insulin resistance plays a role in the phenotype observed, but this was not specifically assessed in the study undertaken. It is of note that ANA has previously been shown to reduce metabolic defects in mouse models of PAH [34]. Metabolic defects have been increasingly associated with PAH clinically. The mechanisms underlying this remain unclear, although ROS have been implicated [38]. Metformin is a well-established drug for use in type 2 diabetes mellitus and has also been proposed as treatment for PAH. We have previously shown metformin can inhibit aromatase expression and activity to decrease oestrogen and its metabolism in experimental pulmonary hypertension [7].

Taken together, these findings suggest that metabolic changes in males during obesity result in the increased synthesis and excretion of 16αOHE1 from VAT that can then act in an endocrine fashion on PASMCs, resulting in ROS generation, oxidative damage and increased proliferation, thus contributing to pulmonary vascular remodelling and the pulmonary hypertension phenotype observed in the animal models studied (supplementary figure S13). The ability of intra-thoracic fat to produce 16αOHE1 lends further weight to this hypothesis given its direct contact with heart and lung tissue. ANA, a third-generation, nonsteroidal, highly selective aromatase inhibitor that is widely used clinically and has been shown to be safe in a small clinical trial in PAH patients [13], had therapeutic effects in both male and female obese mice. The female predisposition to develop PAH is well established [1], mediated partially by the pathogenic effects of oestrogen in the pulmonary circulation [18, 19]. We have demonstrated that endogenous oestrogens can play a role in pulmonary hypertension in males in the presence of modifying factors such as obesity. Obesity may particularly predispose males to the development of PAH due to obesity-mediated VAT dysfunction resulting in altered oestrogen production and metabolism.

## Supplementary material

10.1183/13993003.01524-2018.Supp1 **Please note:** supplementary material is not edited by the Editorial Office, and is uploaded as it has been supplied by the author.
Supplementary material ERJ-01524-2018.Supplement
