# Supplementary material for: Obesity alters oestrogen metabolism and contributes to pulmonary arterial hypertension
Source: Eur Respir J. 2019 Jun 13;53(6):1801524. doi: 10.1183/13993003.01524-2018 (PMC6581204; doi:10.1183/13993003.01524-2018)
Supplement: Supplementary file 1 [file ERJ-01524-2018.Supplement.pdf]

**Online Data Supplement**

*Obesity Alters Oestrogen Metabolism and Contributes to Pulmonary Arterial Hypertension.*

Kirsty M Mair[1], Katie Y Harvey[1], Alasdair D Henry, Dianne Z Hillyard[1], Margaret Nilsen[1], Margaret R. MacLean[1].

[1]Institute of Cardiovascular and Medical Sciences, College of Medical and Veterinary Science, University of Glasgow, Glasgow, UK.

## ***Supplemental Methods***

### *Ethical Information*

All animal procedures conform to the Animals (Scientific Procedures) Act 1986, ARRIVE guidlines and the *Guide for the Care and Use of Laboratory Animals* published by the US National Institutes of Health (NIH publication No. 85–23, revised 1996).

### *Animals*

Male and female C57BL/6JOlaHsd mice, B6.V-Lep$^{ob}$/Lep$^{ob}$/OlaHsd (ob/ob) mice aged 6-8 weeks and their lean litter-mates (B6.V-(lean)/OlaHsd) (aged 6-8 weeks) were obtained from Envigo, UK. Rodents were housed in a 12-hour light-dark cycle with access to food and water ad libitum. Animals were housed together to promote synchronisation of the estrous cycle. All animal experiments and subsequent analysis were carried out in a blinded fashion.

### *Obesity and High fat Diet (HFD)*

We chose to study mice so we could compare transgenically-induced PH with that produced by HFD.

Ob/ob mice are leptin deficient and exhibit obesity from 4-5 weeks of age.

C57BL/6JOlaHsd mice were maintained on normal (BK001 Breeder and Grower diet, Special Diet Services, UK) or HFD (829100 Western RD, percentage calories from fat 42%, protein 15 %, carbohydrate 43%; Special Diet Services, UK) for 20 weeks. During this time, animals were monitored weekly for weight gain and display a

significant increase in body weight over their normal diet controls by 7-weeks post commencement of HFD (Figure S1).

*In Vivo Effects of Anastrozole in obese mice hypoxic PH*

Hypoxia was the model of choice as mice do not develop monocrotailne-induced PH or reproducible robust sugen/hypoxic PH.

The ability of anastrozole to attenuate the development of PH in hypoxic obese ob/ob and HFD mice was assessed. Ob/ob mice and their lean controls were maintained in hypoxia for 14 days to induced PH. During this time they were administered once daily with the aromatase inhibitor, anastrozole (Tocris), 3 $mgkg^{-1}$ or vehicle (1% carboxymethylcellulose) (i.p.). We have previously established that 3 $mgkg^{-1}$ of anastrozole is the optimum therapeutic dose in mice and rats (1). Following 20 weeks on HFD, C57BL/6JOlaHsd mice were also subjected to hypoxia for 14 days and the effects of anastrozole 3 $mgkg^{-1}$ or vehicle, once daily (i.p.), on the development of PH assessed. Age-matched mice housed in normoxic conditions were studied as controls. Both male and female mice were assessed to determine the effects of gender. *n*=6-10 mice per experimental group.

*In Vivo Effects of 2,2',4,6'-tetramethoxystilbene (TMS) in obese mice*

Male ob/ob mice and their lean litter-mates were housed in normoxic conditions for 14 days. During this time the mice were dosed once daily with i.p. injections of the CYP1B1 inhibitor TMS 3 $mgkg^{-1}day^{-1}$ or vehicle (4% ethanol). *n*=8 mice per experimental group were studied.

*Haemodynamic pressure measurements in mice*

Right ventricular systolic pressure was measured by right heart catheterization through the right jugular vein using a 1035 Millar pressure–conductance catheter. Mean systemic arterial pressure was assessed by placement of the catheter in the right carotid artery.  Data was recorded using a MPVS-300 System (Millar, Houston, TX) and LabChart7 Software (ADInstruments, UK).

*Right Ventricular Hypertrophy*

Right ventricular hypertrophy (RVH) in mice was assessed by weight measurement of the right ventricular free wall and left ventricle plus septum. The ratio expressed is RV/LV+S.

*Lung Histopathology*

5µm lung sagittal sections were stained with elastic Van Gieson and pulmonary arteries (<80 µm external diameter) microscopically assessed for degree of muscularisation in a blinded fashion, as previously described (2).  Remodelled arteries were confirmed by the presence of double-elastic laminae.  Briefly, percentage remodelling (percent of remodelled vessels) was defined for each animal by the number of remodelled vessels divided by the total number (≥80 per lung) of vessels observed in the lung. Images were captured using a Zeiss Axio Imager M1.

Right Ventricle Histopathology

5µm transverse sections of the heart were stained with Picrosirius red. Images were captured using an EVOS XL Core Cell Imaging System, Thermofischer, UK and edited using Microsoft Image Composite Editor.

*Measurement of E2, DHEA-S, testosterone and 16αOHE1 ELISA*

Circulating estradiol (E2) was quantified in plasma using a Mouse/Rat Estradiol ELISA (Calbiotech, USA), following the manufacturer's instructions. Plasma DHEA-S levels were also determined using ELISA (Calbiotech, USA). Circulating testosterone levels were quantified in plasma by ELISA following the manufacturer's instructions (R&D systems, UK). Urinary 16αOHE1 was determined by ELISA (Demeditec Diagnostics, Germany).

*Isolation and Culture of Mouse Pulmonary Artery Smooth Muscle cells (mPASMCs)*

mPASMCs wear isolated from third order pulmonary arteries of male lean and ob/ob mice as described previously (3). Cells were cultured in Dulbecco's Modified Eagle Medium (DMEM; Gibco, UK) supplemented with antibiotic antimycotic (AA) solution (containing 0.25µg/ml amphotericin B; 100U/ml penicillin; 100µg/ml streptomycin; Sigma-Aldrich, UK) and 10% (v/v) fetal bovine serum (Sera Laboratories International, West Sussex, UK).

*Preparation of visceral adipose tissue conditioned media (VAT-CM)*

Visceral adipose tissue (VAT) was isolated from ob/ob male mice, washed in PBS and cut in to 2mm chunks. The VAT was then incubated in phenol red free DMEM (no FBS, 5%AA) in a T-75 tissue culture flask for 24 hours at 37$^{o}$C in 5% $CO_2$ in the presence or absence of ANA (100nM) or TMS (100nM). During this time the flask was gently shaken at regular intervals. Following the 24-hour incubation the resultant VAT-CM was filtered using filtered suing a 100µm cell strainer to remove any debris, aliquoted, and stored at -80$^{o}$C. Cell culture media was incubated with visceral adipose

tissue at a ratio of 1g of tissue per 10ml of media. VAT-CM was diluted 1:3 prior to use.

*Assessment of Cell Proliferation*

Cells were grown to 60% confluence in 12-well plates before serum deprivation for 24 hours in 1% fetal bovine serum in phenol-red free DMEM prior to experiments to render them quiescent. Following stimulation, cells were washed, trypsinized and the cell pellet collected by centrifugation at 4900G for 3 minutes. The pellet was then resuspended in 15µl PBS. Immediately prior to counting 15 µl of 0.4% trypan blue solution was added to the suspension and the number of live cells assessed using a Countess II FL cell counter, Life Technologies, UK.

*Quantitative Reverse Transcription–Polymerase Chain Reaction*

mRNA expression was assessed in the lungs, VAT and RV of mice by quantitative reverse transcription–polymerase chain reaction. At necropsy, tissue from each rodent was removed and snap frozen. RNA was then extracted from the tissue and gene expression quantified using TaqMan® gene expression assays (assay details shown in online supplemental Table S1). ViiA7 Real-time PCR system (Applied Biosystems) was programmed for PCR conditions 95oC for 10 minutes followed by 50 cycles of 95o C for 15 seconds and 60o C for 1 minute. Results were normalised to TATA-binding protein (tbp). The fold change for every gene was obtained using the 2-ΔΔCt method and expressed relative to lean control as appropriate.

*Immunoblotting*

Protein expression was assessed in whole lung and mPASMC lysates. Briefly, lung tissue was homogenised in radioimmunoprecipitation assay (RIPA) buffer via homogenization using a TissueLyser II, Quiagen, UK. mPASMCs were grown in 6 well plates. Following stimulation, cell culture media was removed, the cell mon-layer washed and RIPA buffer added prior to scraping of cells from the well. Samples were denatured and electrophoresed on SDS-PAGE gel. The protein concentrations of each sample were determined by BCA assay and equal concentrations for each sample were loaded per well. Separated proteins were transferred to PVDF membrane and incubated for 1 hour with 5% milk/TBST (w/v) before incubating overnight at 4$^{o}$C with antibodies against aromatase, CYP1B1 and GAPDH. Aromatase, CYP1B1, PCNA and GAPDH molecular weights were detected at 55, 61, 29 and 39 kDa, respectively. Densitometric analysis was performed with TotalLab TL100 software. Data are expressed relative to GAPDH density. Representative immunoblots are cropped from full length blots. For details of primary antibodies used *see* Table S2. In some instances, blots were initially probed for one protein of interest then subsequently stripped and re-probed for other proteins including loading controls.

*Protein tyrosine phosphatase oxidation (Oxy-PTP) assessment*

Irreversible oxidation of protein tyrosine phosphatases (PTPs) was assessed by immunoblotting using an antibody that specifically recognizes the sulfonic acid form of PTP cysteine residues (anti-Ox-PTP; 1:2000 dilution, R&D Systems). Following treatment, the sample is alkylated using N-ethylmaleimide (NEM; 1mmol/L) to protect reduced cysteines. The sulfonic acid form is detected using the Ox-PTP antibody by immunoblotting as previously described (4).

*Reactive Oxygen Species Determination in Lung Sections by Immunofluorescence*

Immunohistochemistry of the reactive oxygen species (ROS) marker 8-hydroxyguanosine (8-OHG) was determined in the lungs of lean and ob/ob male and female mice and chow and HFD fed male and female mice, treated with aromatase inhibitor, anastrozole, or vehicle. Briefly, sections were deparaffinised in a xylene-ethanol-water gradient. Antigen retrieval was performed by boiling slides in EDTA (pH 8.0) for 15 minutes before a 1-hour incubation in a humidified chamber at room temperature with 10% donkey serum/1% bovine serum albumin in 1× TBS-T to block non-specific binding. Sections were incubated with anti- 8-OHG overnight at 4°C (5 μg/mL, ab10802, Abcam). For identification of 8-OHG-positive cells, sections were incubated with Alexa-fluor-488-conjugated donkey anti-goat secondary antibody (A-11055, Molecular Probes, Life Technologies, UK) for 45 minutes at room temperature in the dark. Lipofuscin-mediated auto-fluorescence was removed with 0.1% Sudan Black B (Sigma-Aldrich) in methanol w/v for 10 minutes. Slides were mounted with Pro-Long Gold anti-fade mounting media containing DAPI (4′,6-diamidino-2-phenylindole; P-36931, Molecular Probes, Life Technologies). Fluorescence imaging was measured in an Axiovert 200M microscope with a laser-scanning module LSM 510 (Carl Zeiss, Germany). DAPI was excited at 405nm and Alexa-Fluor 488 at 488nm. Images were captured with the LSM 510 Zen evaluation software (Zeiss, UK).

**Figure S1.** Weight gain of normal diet and high fat diet fed (HFD) mice. Male C57BL/6JOlaHsd mice were maintained on normal (BK001 Breeder and Grower diet, Special Diet Services, UK) or HFD (829100 Western RD, percentage calories from fat 42%, protein 15 %, carbohydrate 43%; Special Diet Services, UK) for 20 weeks. Graph shows weight gain in these animals over a 20 week period. *n=10* per group. * p<0.05, **p<0.01, ***p<0.001, as determined by two-way ANOVA with Bonferroni post-hoc tests.

**Figure S2.** Characterisation of aromatase expression in visceral adipose tissue (VAT) from HFDmice. Representative immunoblots and quantification of aromatase protein expression in VAT from (*a*) maleand (b) female lean and high fat diet fed (HFD) mice. Data are displayed as mean ± SEM, *n*=3 per group. ** p<0.01 as determined by unpaired, two-tailed test. GAPDH = glyceraldehyde phosphate dehydrogenase.

**Figure S3.** Effects of aromatase inhibition on mean systemic arterial pressure (mSAP), left ventricle plus septum (LV+S) weight and LVEDP in ob/ob . **Male mice:** Effects of the aromatase inhibitor, anastrozole (ANA), 3 $mgkg^{-1}day^{-1}$ for 14 days on (a) mSAP, (*b*) LV+S weight and (c) left ventricular end diastolic pressure (LVEDP) in male lean and ob/ob mice. **Female mice:** Effects of the aromatase inhibitor, anastrozole (ANA), 3 $mgkg^{-1}day^{-1}$ for 14 days on (d) mSAP, (*e*) LV+S weight and (f) LVEDP in female lean and ob/ob mice. Data are displayed as mean ± SEM, (n=3-6 per group).

**Figure S4.** Effect of obesity and anstrozole (ANA) treatment on markers of fibrosis and heart failure in the right ventricle (RV). Relative gene expression levels of (a) connective tissue growth factor (CTFG), (b) fibronectin (FN1), (c) natriuretic peptide type A (NPPA) and (d) natriuretic peptide type B (NPPB) in RV tissue male lean and ob/ob mice treated with or without anastrozole. (e) Picrosirius Red staining in RV of male and female lean and ob/ob mice treated with and without ANA. Images shown are representative. Scale bar (-) represents 500µm. Gene expression levels are normalized to TATA-binding protein (tbp). All data are expressed as mean ± SEM (n=3-5 per group).

**Figure S5.** Inhibition of aromatase attenuates parameters of pulmonary hypertension in high fat diet fed (HFD)mice. **Male:** Effects of the ANA, 3 $mgkg^{-1}day^{-1}$ for 14 days on (*a*) RVSP (n=5-10 per group), (*b*) RVH (n=5-10 per group) (as determined by RV/LV+S ratio) and (*c*) the % of remodelled pulmonary arteries (n=5 per group) in normoxic and hypoxic male HFD mice. (*d*) Representative images of pulmonary arteries from normoxic and hypoxic male HFDmice treated with or without anastrozole, 3 $mgkg^{-1}day^{-1}$ (Scale bar (**-**) = 50µm). **Female:** Effects of the ANA, 3 $mgkg^{-1}day^{-1}$ for 14 days on (*e*) RVSP (n=5-7 per group), (f) RVH (n=5-7 per group) (as determined by RV/LV+S ratio) and (*g*) the % of remodeled pulmonary arteries (n=5 per group in normoxic and hypoxic female HFD fed mice. (*h*) Representative images of pulmonary arteries from normoxic and hypoxic female HFD mice treated with or without anastrozole, 3 $mgkg^{-1}day^{-1}$ (Scale bar (**-**) = 50µm). Data are displayed as mean ± SEM. *p<0.05, ** p<0.01 and *** p<0.001 as indicated, determined by one-way ANOVA with Bonferroni post-test. RV/LV+S = right ventricle/left ventricle + septum.

**Figure S6.** Effects of aromatase inhibition on mean systemic arterial pressure (mSAP), left ventricle plus septum (LV+S) weight and LVEDP in high fat diet fed (HFD)

mice . **Male mice:** Effects of the aromatase inhibitor, anastrozole (ANA), 3 $mgkg^{-1}day^{-1}$ for 14 days on (a) mSAP, (*b*) LV+S weight and (c) left ventricular end diastolic pressure (LVEDP) in male lean and HFD mice. **Female mice:** Effects of the aromatase inhibitor, anastrozole (ANA), 3 $mgkg^{-1}day^{-1}$ for 14 days on (d) mSAP, (*e*) LV+S weight and (f) LVEDP in female lean and HFD mice. Data are displayed as mean ± SEM, (n=3-6 per group).

**Figure S7.** *Effects of aromatase inhibition on body weight in ob/ob and high fat diet fed (HFD) mice.* **Male mice:** Effects of the aromatase inhibitor, anastrozole (ANA), 3 $mgkg^{-1}day^{-1}$ for 14 days on body weight in (*a*) ob/ob and (*b*) HFD fed mice (*n*= 5-10 per group). **Female mice:** Effects of the aromatase inhibitor, anastrozole (ANA), 3 $mgkg^{-1}day^{-1}$ for 14 days on body weight in (c) ob/ob and (*d*) HFD fed mice (*n*=5-10 per group). Data are displayed as mean ± SEM. *p<0.05 and ***p<0.001 as indicated, determined by one-way ANOVA with Bonferroni post-hoc test. **Figure S8.** *Plasma oestrogen (E2) in high fat diet fed (HFD)mice.*

*Circulating plasma E2 levels in (a) male(n= 5-10 per group),and (b) female normoxic and hypoxic lean and HFD fed mice (n=5 per group) treated with or without anastrozole 3 $mgkg^{-1}day^{-1}$.* Data are displayed as mean ± SEM. *p<0.05 and ***p<0.001 as indicated, determined by one-way ANOVA with Bonferroni post-hoc test.

**Figure S9**. *Uterus weights and testosterone levels in obese mice. Uterus weights in (a) normoxic lean and ob/ob mice (n= 5-10 per group) and (d) hypoxic lean and HFD fed mice (n=4-8 per group) treated with or without anastrozole 3 $mgkg^{-1}day^{-1}$.* Circulating plasma testosterone levels in (c) normoxic and hypoxic lean and ob/ob mice (n=3-8 per group) and (d) normoxic and hypoxic lean and HFD fed mice (n=5-9 per group) treated with or without anastrozole 3 $mgkg^{-1}day^{-1}$. Data are displayed as

mean ± SEM. *p<0.05, ** p<0.01 and *** p<0.001 as indicated, determined by one-way ANOVA with Bonferroni post-test.

**Figure S10.** Characterisation of changes in CYP1B1 expression in visceral adipose tissue (VAT) and 16α-hydroxyestrone (16αOHE1) levels in lean *versus* high fat diet fed (HFD )mice. Representative immunoblot and quantification of CYP1B1 protein expression in VAT from (*a*) male and (b) female normal diet and high fat diet (HFD) mice. Data are displayed as mean ± SEM, *n*=3-4 per group. * p<0.05 as indicated, determined by two-tailed, unpaired *t* test GAPDH = glyceraldehyde phosphate dehydrogenase. Urinary 16αOHE1 levels in (*c*) maleand (d) female normal diet and HFD mice. Data are displayed as mean ± SEM, (n=5-7 per group). ** p<0.01 and *** p<0.001 as indicated, determined by one-way ANOVA with Bonferroni post-test.

**Figure S11.** Effects of aromatase inhibition on obesity-induced changes in oxidant and antioxidant damage in mouse lung. (a) Relative gene expression levels of (a) superoxide dismutase 1 (SOD1), (b) catalase and (c) NOX4 in whole lung from male lean and ob/ob mice treated with or without anastrozole. Relative gene expression levels of (d) superoxide dismutase (SOD1) and € catalase in whole lung from female lean and ob/ob mice treated with or without anastrozole. Gene expression levels are normalised to TATA-binding protein (tbp). All data are expressed as mean ± SEM (n=3-8 per group). *p<0.05, ** p<0.01 and *** p<0.001 as indicated, determined by one-way ANOVA with Bonferroni post-test of normoxic and hypoxic groups independently.

**Figure S12.** Expression of antioxidant enzymes in visceral adipose tissue (VAT) from male lean and obese mice. Relative gene expression levels of (a) superoxide dismutatse 1 (SOD1), (b) catalase and (c) glutathione peroxidae (GPX1) in VAT from

male lean and ob/ob normoxic mice treated with or without anastrozole (ANA). Gene expression levels are normalised to TATA-binding protein (tbp). All data are expressed as mean ± SEM (n=5-6 per group). ** $p<0.01$ as indicated, determined by one-way ANOVA with Bonferroni post-test.

**Figure S13.**

Summary of results in high fat diet obese mice. Where inhibition by anastrozole (ANA) is indicated this suggests dependency on endogenous oestrogen (E2). Pulmonary hypertension (PH) was not evident in normoxic lean (normal diet) males or females. Obese males and females developed significantly more pulmonary vascular remodelling (PVR) than lean males. In the hypoxic male obese mice, endogenous E2 contributed to elevations in all parameters of PH in males. In females, ANA only decreased right ventricular systolic pressure (RVSP) and had no effect on right ventricular hypertrophy (RVH) and PVR. There was a decrease in plasma E2 with an increase in urinary 16αOHE1 in male HFD obese mice and visceral adipose tissue (VAT) aromatase and CYP1B1 expression was increased.

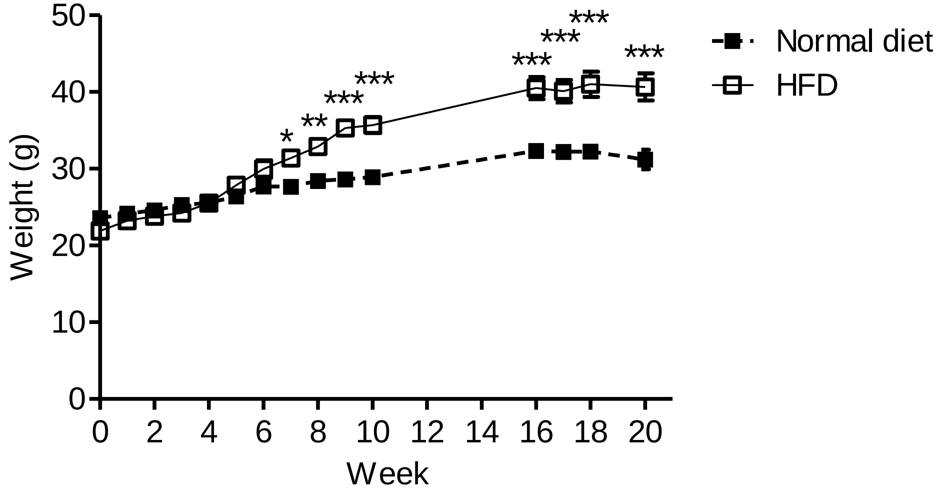
50
40
30
20
10
0
Weight (g)
0
2
4
6
8
10
12
14
16
18
20
Week
*
**
***
***
***
***
***
***
Normal diet
HFD

Figure S1

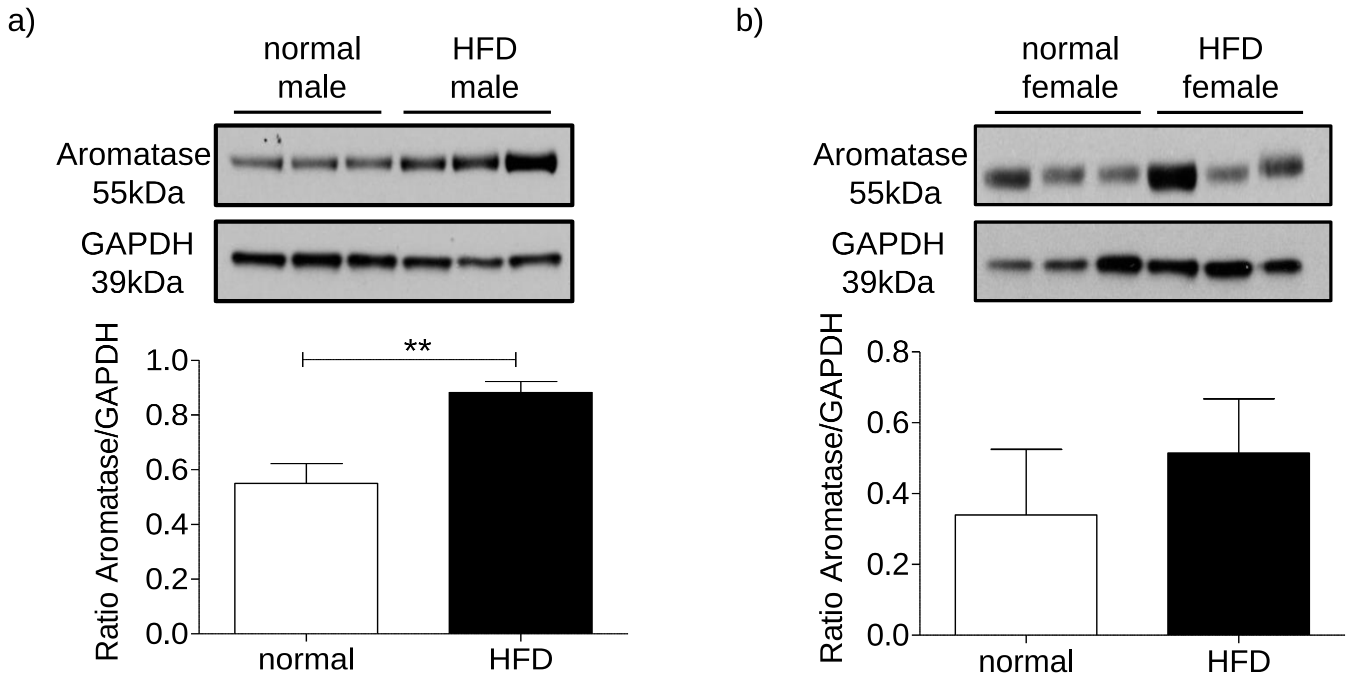
a)
normal male
HFD male
Aromatase 55kDa
GAPDH 39kDa
Ratio Aromatase/GAPDH
1.0
0.8
0.6
0.4
0.2
0.0
**
normal
HFD
b)
normal female
HFD female
Aromatase 55kDa
GAPDH 39kDa
Ratio Aromatase/GAPDH
0.8
0.6
0.4
0.2
0.0
normal
HFD

Figure S2

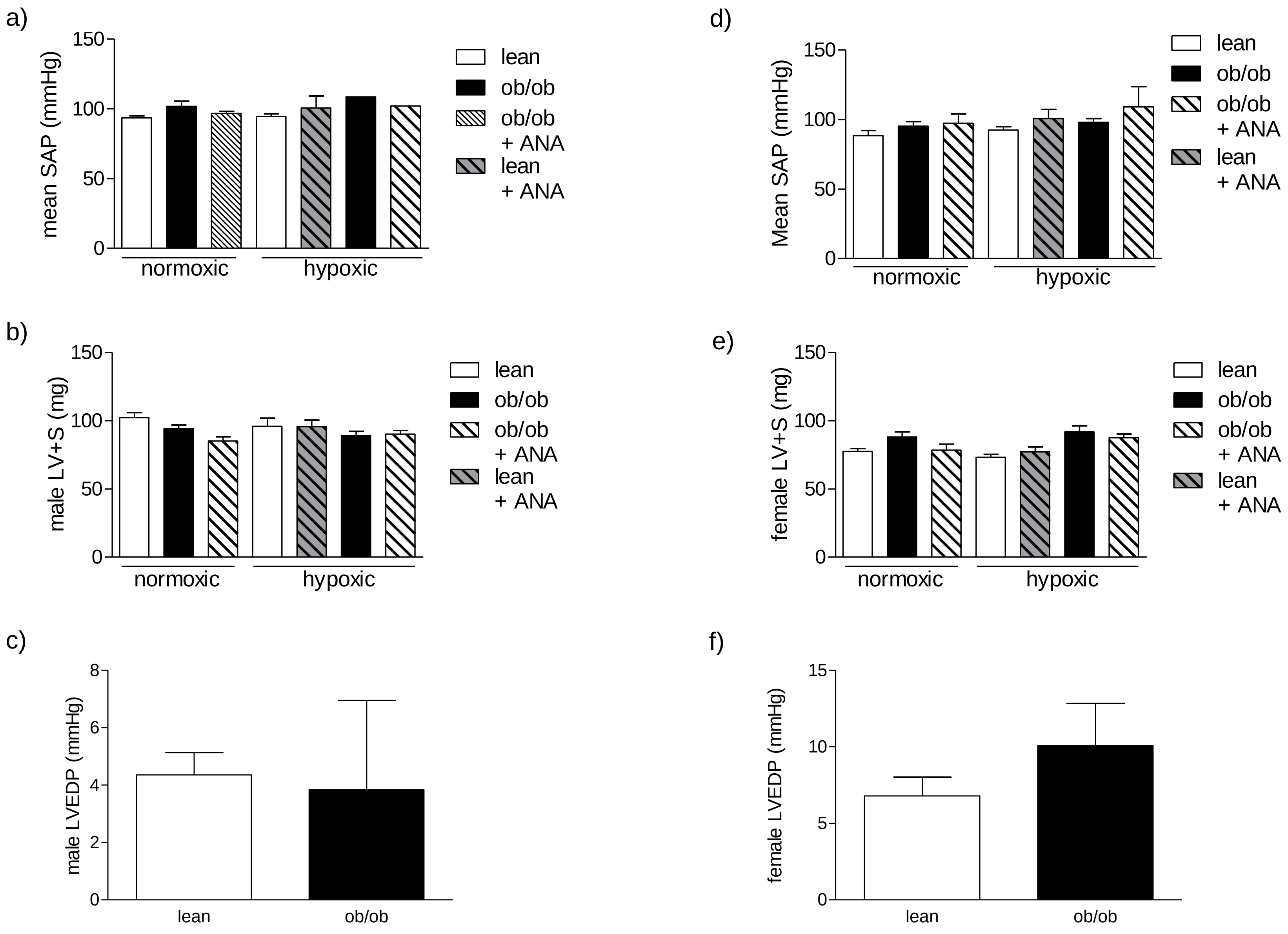
a)
mean SAP (mmHg)
150
100
50
0
normoxic
hypoxic
lean
ob/ob
ob/ob + ANA
lean + ANA
d)
Mean SAP (mmHg)
150
100
50
0
normoxic
hypoxic
lean
ob/ob
ob/ob + ANA
lean + ANA
b)
male LV+S (mg)
150
100
50
0
normoxic
hypoxic
lean
ob/ob
ob/ob + ANA
lean + ANA
e)
female LV+S (mg)
150
100
50
0
normoxic
hypoxic
lean
ob/ob
ob/ob + ANA
lean + ANA
c)
male LVEDP (mmHg)
8
6
4
2
0
lean
ob/ob
f)
female LVEDP (mmHg)
15
10
5
0
lean
ob/ob

Figure S3

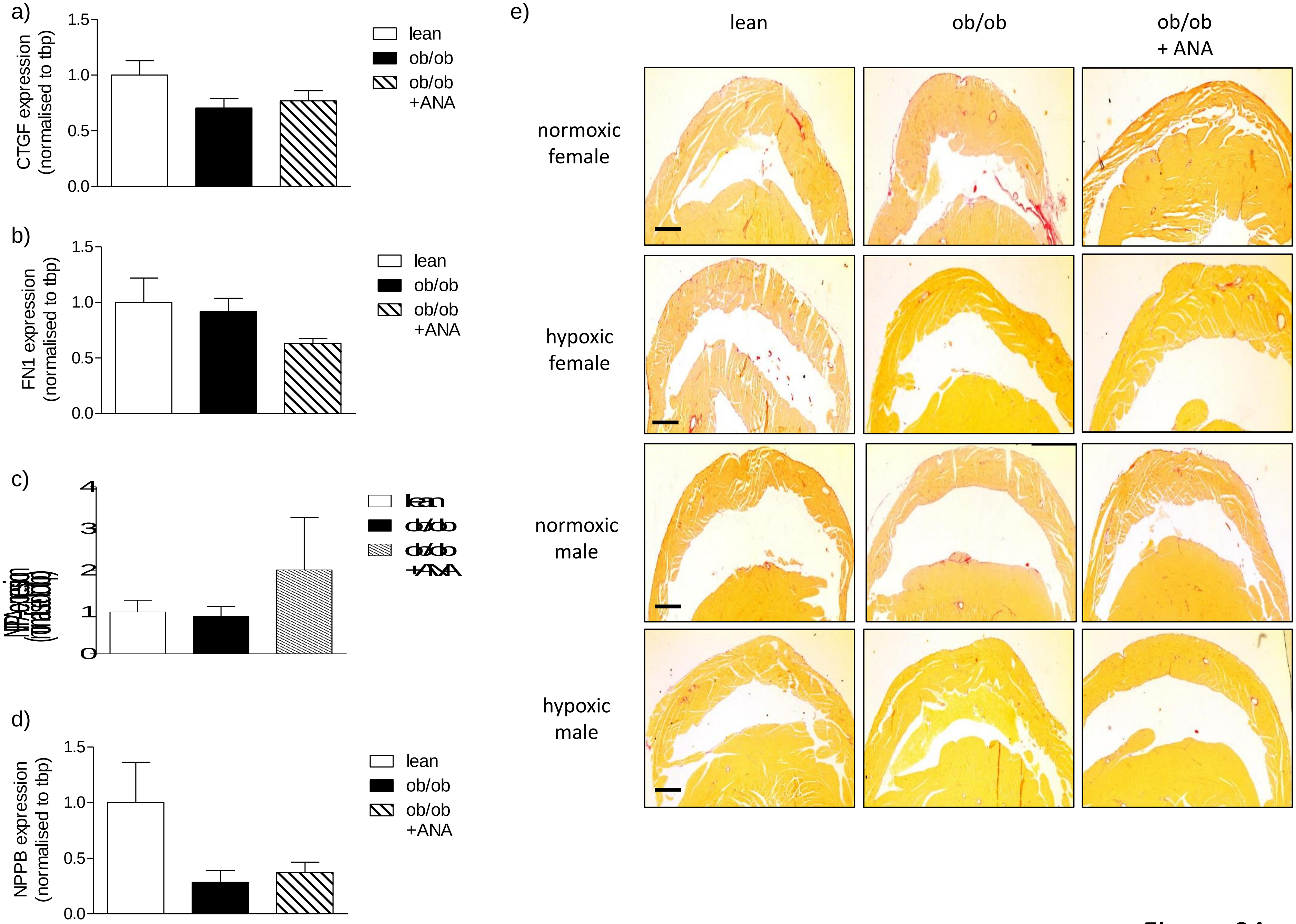
a)
CTGF expression
(normalised to tbp)
1.5
1.0
0.5
0.0
lean
ob/ob
ob/ob
+ANA
b)
FN1 expression
(normalised to tbp)
1.5
1.0
0.5
0.0
lean
ob/ob
ob/ob
+ANA
c)
4
3
2
1
0
d)
NPPB expression
(normalised to tbp)
1.5
1.0
0.5
0.0
lean
ob/ob
ob/ob
+ANA
e)
lean
ob/ob
ob/ob
+ ANA
normoxic
female
hypoxic
female
normoxic
male
hypoxic
male

Figure S4

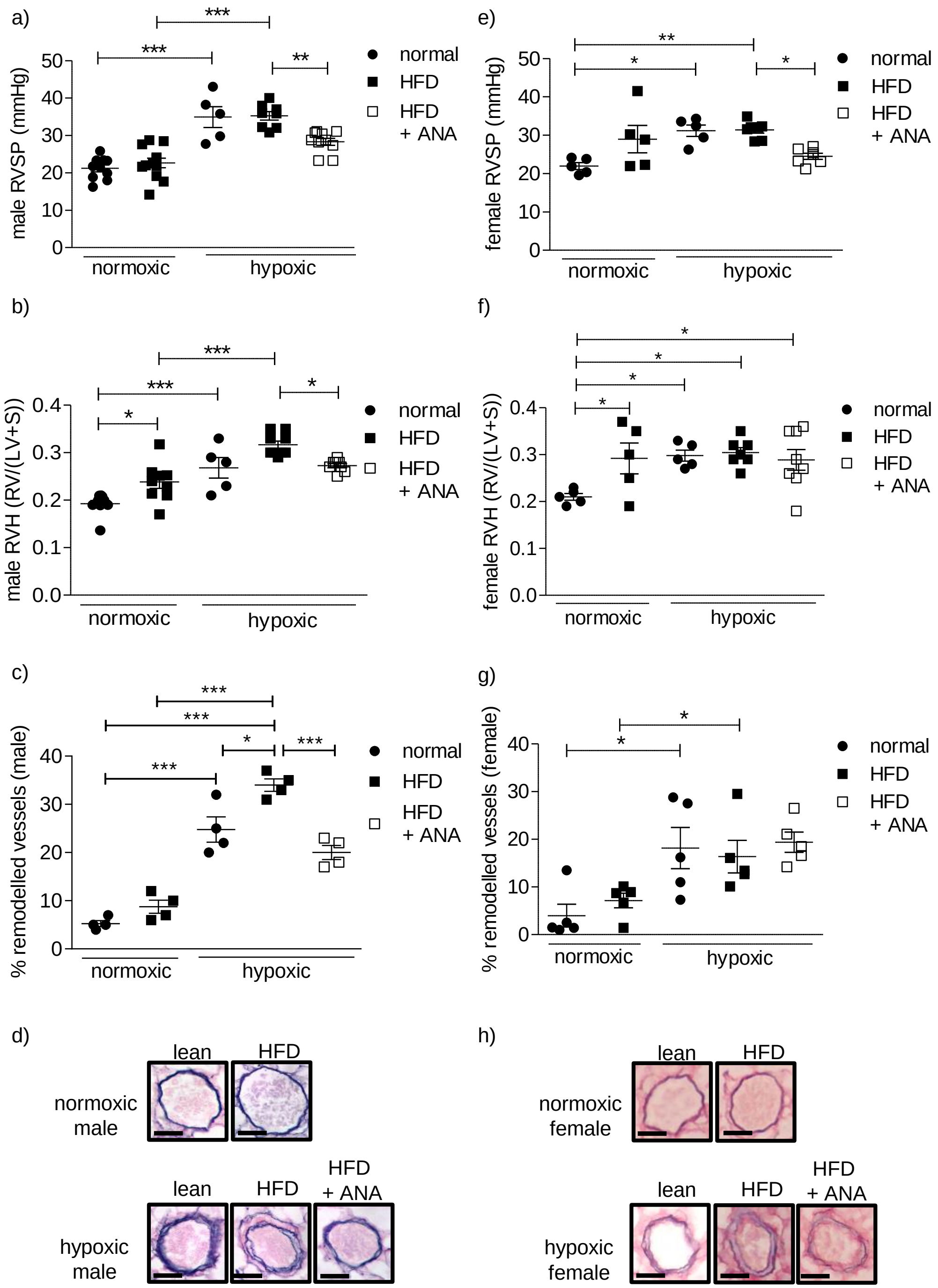
a)
male RVSP (mmHg)
normoxic
hypoxic
normal
HFD
HFD + ANA
b)
male RVH (RV/(LV+S))
c)
% remodelled vessels (male)
d)
normoxic male
hypoxic male
lean
HFD
HFD + ANA
e)
female RVSP (mmHg)
f)
female RVH (RV/(LV+S))
g)
% remodelled vessels (female)
h)
normoxic female
hypoxic female

Figure S5

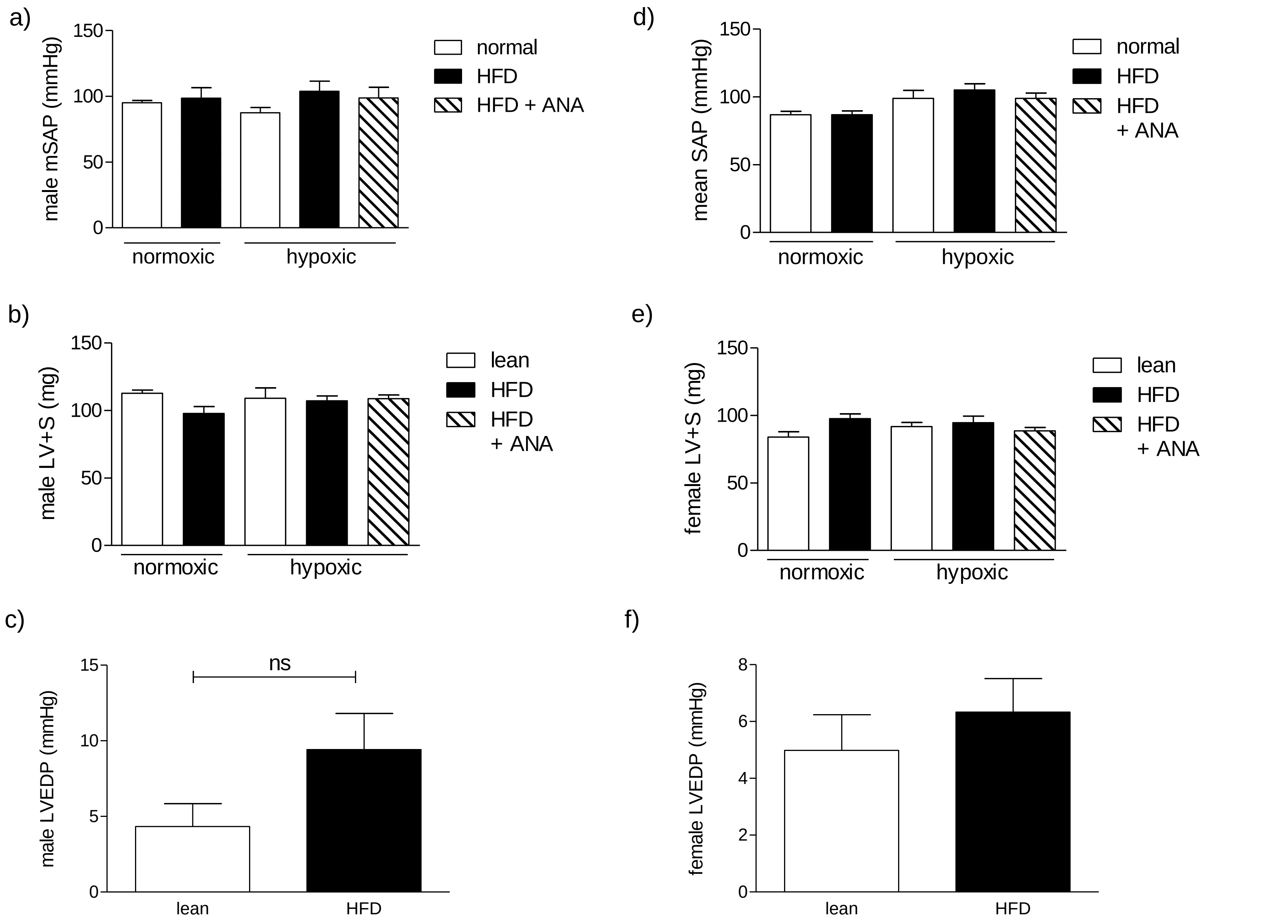

a)
male mSAP (mmHg)
150
100
50
0
normoxic
hypoxic
normal
HFD
HFD + ANA
b)
male LV+S (mg)
150
100
50
0
normoxic
hypoxic
lean
HFD
HFD
+ ANA
c)
male LVEDP (mmHg)
15
10
5
0
ns
lean
HFD
d)
mean SAP (mmHg)
150
100
50
0
normoxic
hypoxic
normal
HFD
HFD
+ ANA
e)
female LV+S (mg)
150
100
50
0
normoxic
hypoxic
lean
HFD
HFD
+ ANA
f)
female LVEDP (mmHg)
8
6
4
2
0
lean
HFD


Figure S6

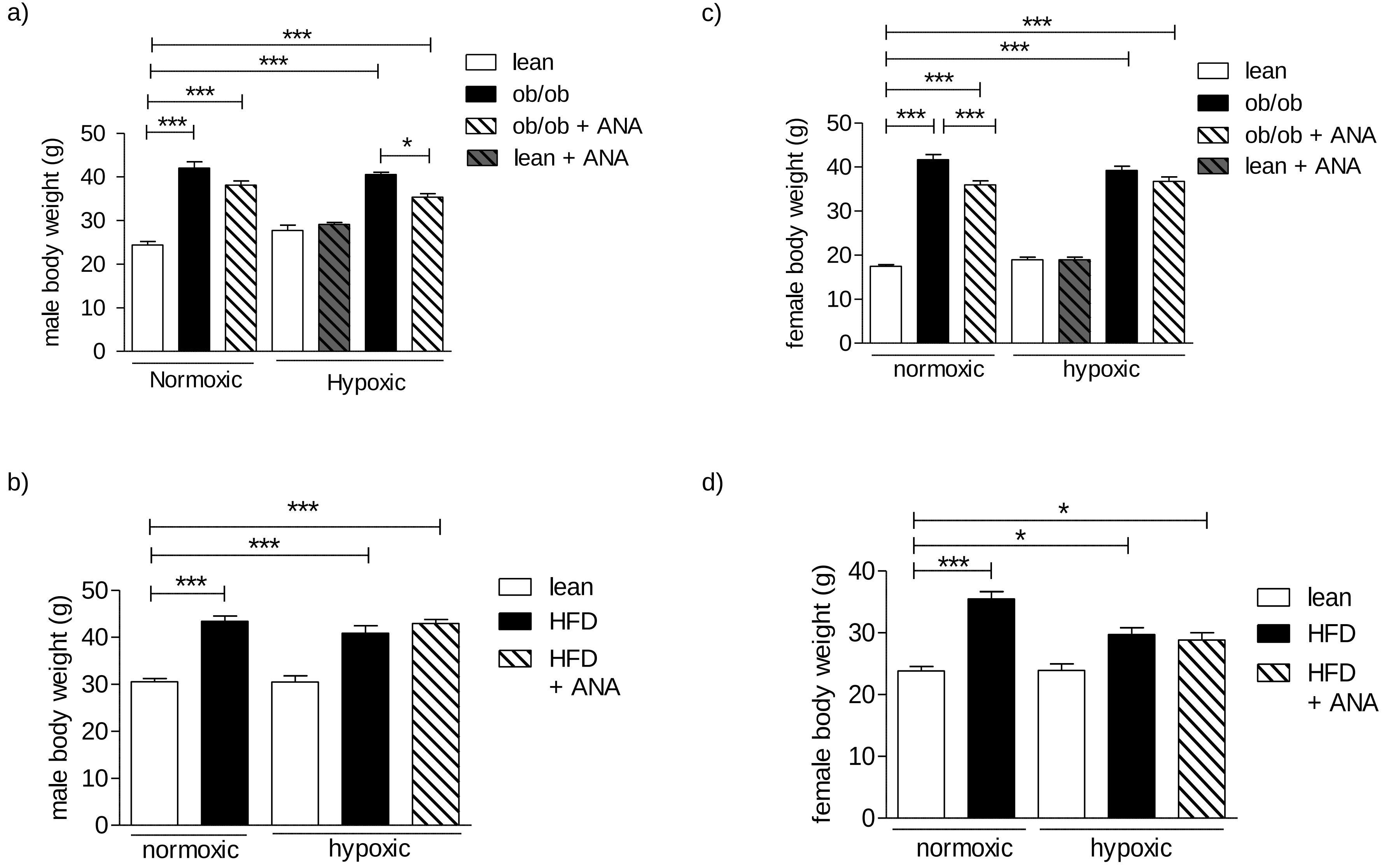
a)
***
***
***
***
*
lean
ob/ob
ob/ob + ANA
lean + ANA
male body weight (g)
50
40
30
20
10
0
Normoxic
Hypoxic
c)
***
***
***
***
***
lean
ob/ob
ob/ob + ANA
lean + ANA
female body weight (g)
50
40
30
20
10
0
normoxic
hypoxic
b)
***
***
***
lean
HFD
HFD
+ ANA
male body weight (g)
50
40
30
20
10
0
normoxic
hypoxic
d)
*
*
***
lean
HFD
HFD
+ ANA
female body weight (g)
40
30
20
10
0
normoxic
hypoxic

Figure S7

a)

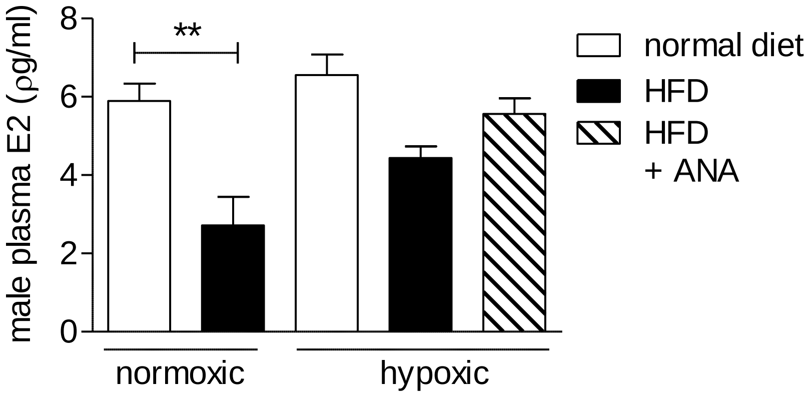

male plasma E2 (ρg/ml)
8
6
4
2
0
**
normal diet
HFD
HFD
+ ANA
normoxic
hypoxic


b)

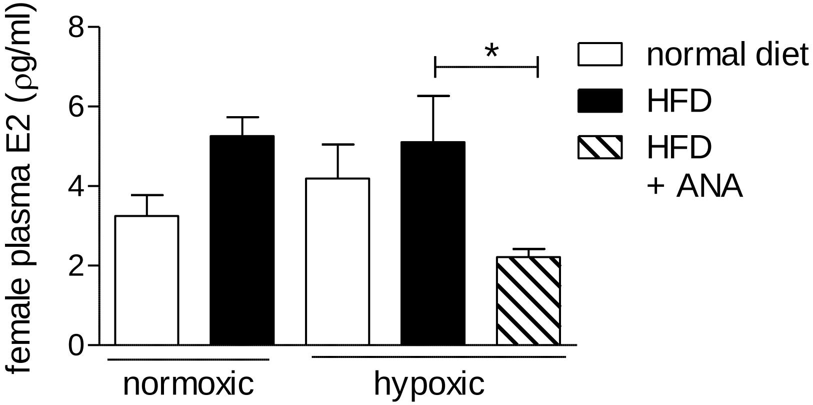

female plasma E2 (ρg/ml)
8
6
4
2
0
*
normal diet
HFD
HFD
+ ANA
normoxic
hypoxic


Figure S8

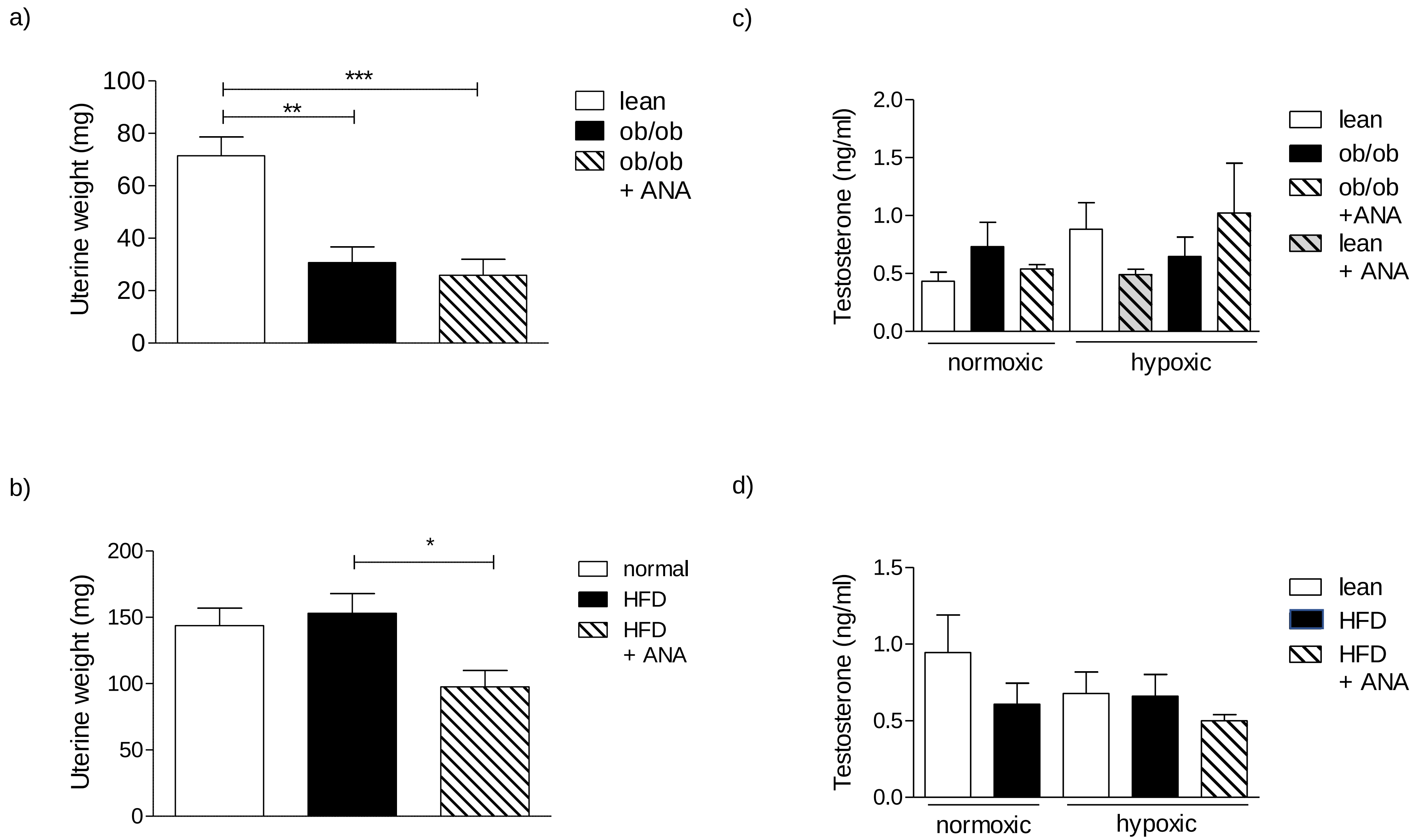
a)
Uterine weight (mg)
100
80
60
40
20
0
***
**
lean
ob/ob
ob/ob
+ ANA
b)
Uterine weight (mg)
200
150
100
50
0
*
normal
HFD
HFD
+ ANA
c)
Testosterone (ng/ml)
2.0
1.5
1.0
0.5
0.0
normoxic
hypoxic
lean
ob/ob
ob/ob
+ANA
lean
+ ANA
d)
Testosterone (ng/ml)
1.5
1.0
0.5
0.0
normoxic
hypoxic
lean
HFD
HFD
+ ANA

Figure S9

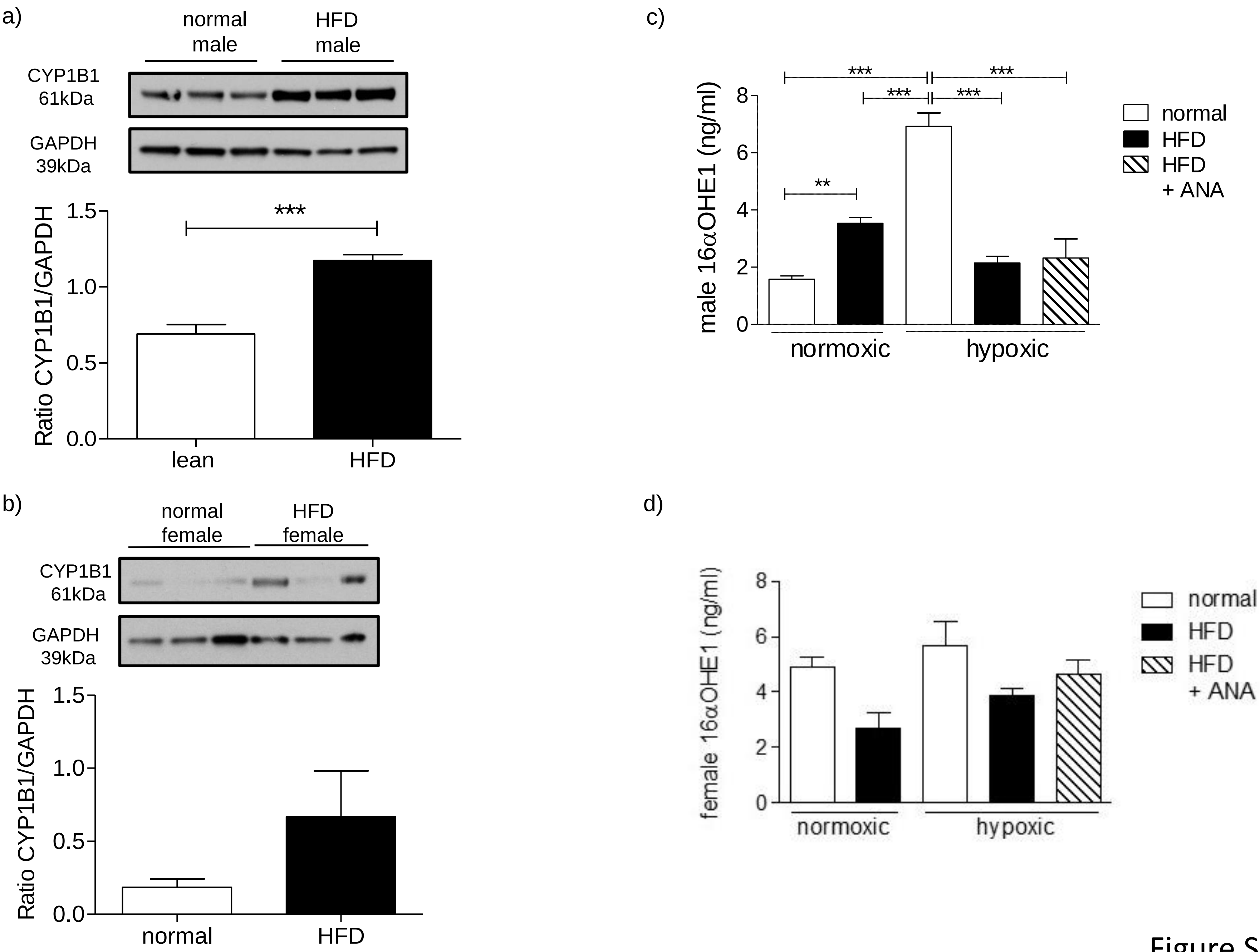

a)
normal male
HFD male
CYP1B1 61kDa
GAPDH 39kDa
Ratio CYP1B1/GAPDH
1.5
1.0
0.5
0.0
***
lean
HFD
b)
normal female
HFD female
CYP1B1 61kDa
GAPDH 39kDa
Ratio CYP1B1/GAPDH
1.5
1.0
0.5
0.0
normal
HFD
c)
male 16αOHE1 (ng/ml)
8
6
4
2
0
***
***
***
***
**
normoxic
hypoxic
normal
HFD
HFD + ANA
d)
female 16αOHE1 (ng/ml)
8
6
4
2
0
normoxic
hypoxic
normal
HFD
HFD + ANA


Figure S10

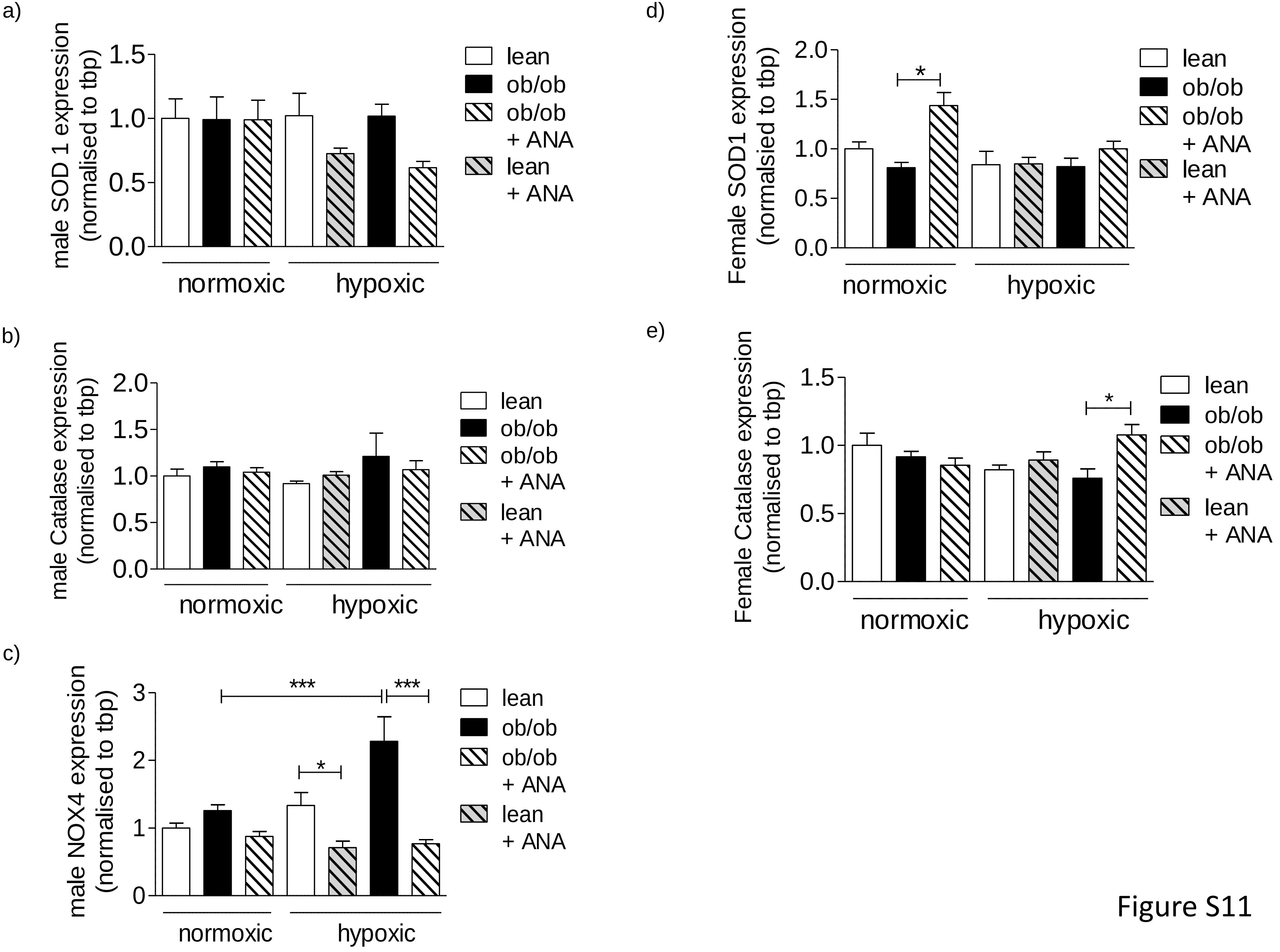
a)
male SOD 1 expression (normalised to tbp)
1.5
1.0
0.5
0.0
normoxic
hypoxic
lean
ob/ob
ob/ob + ANA
lean + ANA
b)
male Catalase expression (normalised to tbp)
2.0
1.5
1.0
0.5
0.0
normoxic
hypoxic
lean
ob/ob
ob/ob + ANA
lean + ANA
c)
male NOX4 expression (normalised to tbp)
3
2
1
0
***
***
*
normoxic
hypoxic
lean
ob/ob
ob/ob + ANA
lean + ANA
d)
Female SOD1 expression (normalsied to tbp)
2.0
1.5
1.0
0.5
0.0
*
normoxic
hypoxic
lean
ob/ob
ob/ob + ANA
lean + ANA
e)
Female Catalase expression (normalised to tbp)
1.5
1.0
0.5
0.0
*
normoxic
hypoxic
lean
ob/ob
ob/ob + ANA
lean + ANA

Figure S11

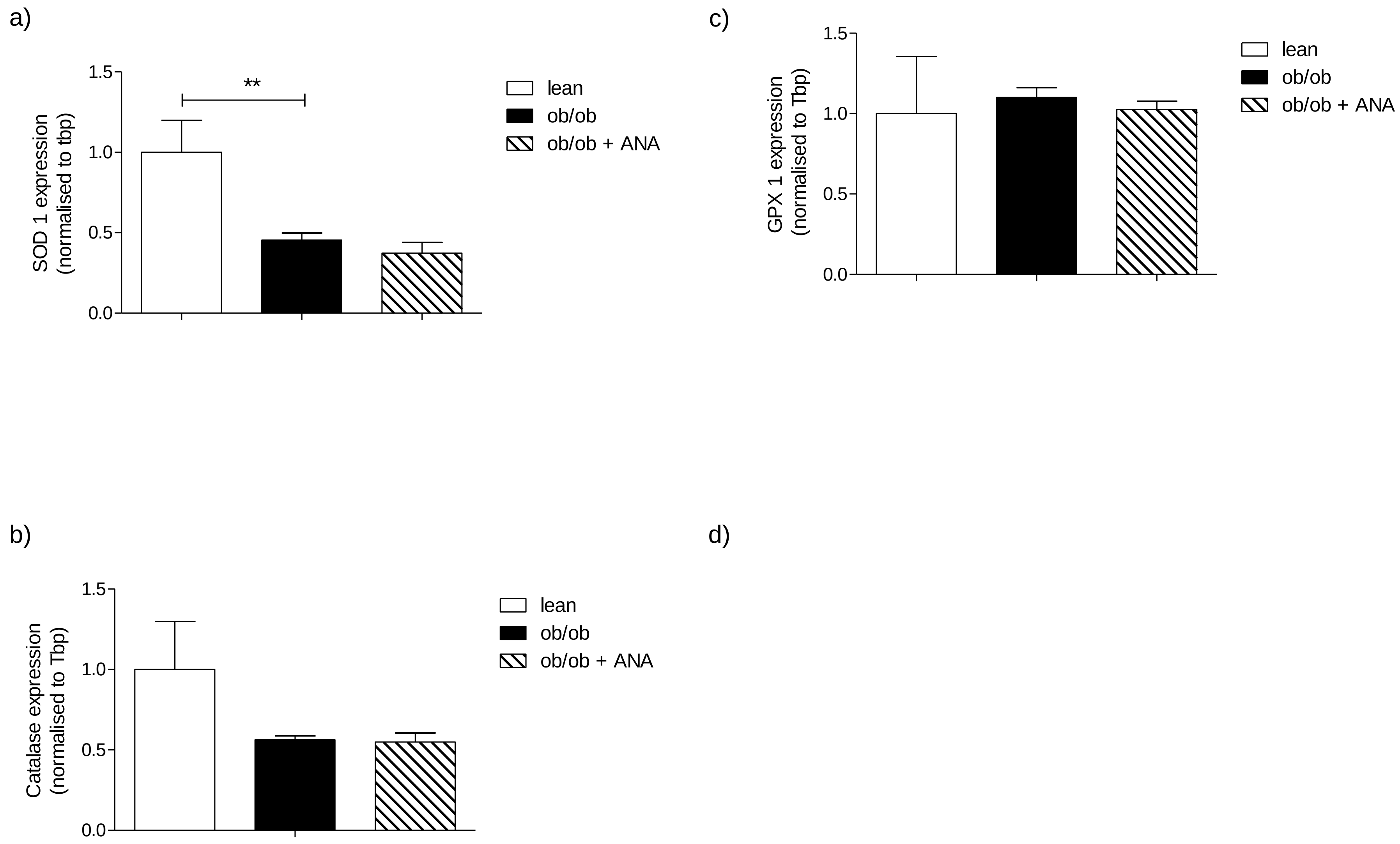

a)
1.5
1.0
0.5
0.0
SOD 1 expression
(normalised to tbp)
**
lean
ob/ob
ob/ob + ANA
c)
GPX 1 expression
(normalised to Tbp)
b)
Catalase expression
(normalised to Tbp)


d)

Figure S12

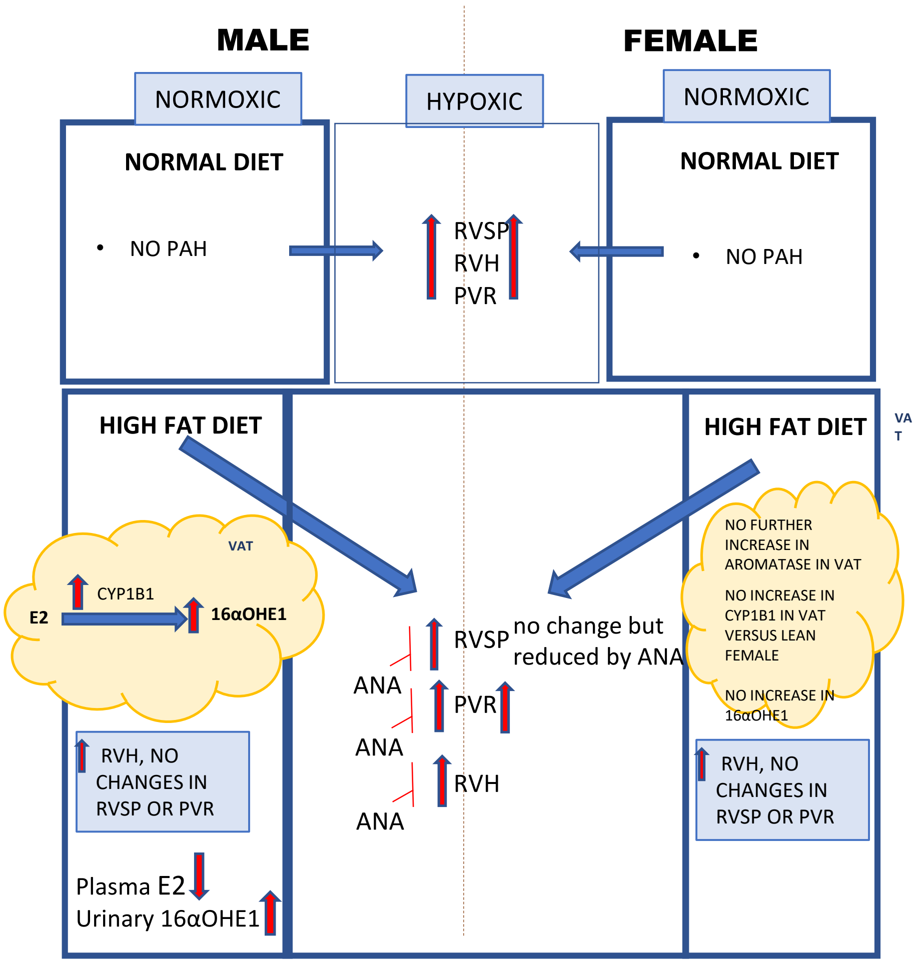
MALE
FEMALE
NORMOXIC
HYPOXIC
NORMOXIC
NORMAL DIET
NO PAH
RVSP
RVH
PVR
NORMAL DIET
NO PAH
HIGH FAT DIET
HIGH FAT DIET
VAT
VAT
CYP1B1
E2
16αOHE1
RVSP
no change but reduced by ANA
ANA
PVR
ANA
RVH
ANA
NO FURTHER INCREASE IN AROMATASE IN VAT
NO INCREASE IN CYP1B1 IN VAT VERSUS LEAN FEMALE
NO INCREASE IN 16αOHE1
RVH, NO CHANGES IN RVSP OR PVR
RVH, NO CHANGES IN RVSP OR PVR
Plasma E2
Urinary 16αOHE1

Figure S13

***References***

1. Mair KM, Wright AF, Duggan N, Rowlands DJ, Hussey MJ, Roberts S*, et al.* Sex-dependent influence of endogenous estrogen in pulmonary hypertension. *Am J Respir Crit Care Med* 2014; 190: 456-467.

2. Keegan A, Morecroft I, Smillie D, Hicks MN, MacLean MR. Contribution of the 5-HT(1B) receptor to hypoxia-induced pulmonary hypertension: converging evidence using 5-HT(1B)-receptor knockout mice and the 5-HT(1B/1D)-receptor antagonist GR127935. *Circulation research* 2001; 89: 1231-1239.

3. Hood KY, Mair KM, Harvey AP, Montezano AC, Touyz RM, MacLean MR. Serotonin Signaling Through the 5-HT1B Receptor and NADPH Oxidase 1 in Pulmonary Arterial Hypertension. *Arteriosclerosis, thrombosis, and vascular biology* 2017; 37: 1361-1370.

4. Persson C, Kappert K, Engstrom U, Ostman A, Sjoblom T. An antibody-based method for monitoring in vivo oxidation of protein tyrosine phosphatases. *Methods (San Diego, Calif)* 2005; 35: 37-43.

| ***Gene*** | ***Assay ID*** |
|---|---|
| ***Catalase*** | *Mm00437992_m1* |
| ***Connective tissue growth factor (CTGF)*** | *Mm01192933_g1* |
| ***Fibronectin (FN-1)*** | *Mm01256744_m1* |
| ***Glutathione Peroxidase (Gpx1)*** | *Mm00656767_g1* |
| ***Nox4*** | *Mm00479246_m1* |
| ***NPPA*** | *Mm01255747_g1* |
| ***NPPB*** | *Mm01255770_g1* |
| ***Superoxide Dismutase 1 (SOD1)*** | *Mm1344233_g1* |
| ***TATA binding protein (tbp)*** | *Mm1277042_m1* |

**Table S1.** TaqMan® gene expression assays purchased from Thermofischer, UK.

| Antibody | Type (Clone) | Source (catalogue number) | Dilution used |
|---|---|---|---|
| *Aromatase* | *Rabbit Polyclonal IgG* | *Sigma (SAB4500606)* | *1:1000 in 5%BSA/TBST* |
| *CYP1B1* | *Rabbit monoclonal [EPR14972]* | *Abcam (Ab185954)* | *1:2500 in 5%BSA/TBST* |
| *Oxy-PTP* | *Monoclonal Mouse IgG1 (Clone # 335636)* | *R&D sytems (MAB2844)* | *1:1000 in 2%Milk/TBST* |
| *GAPDH* | *Monoclonal Mouse [mAbcam 8484]* | *Abcam (Ab9482)* | *1:10,000 in 5%BSA/TBST* |
| *PCNA* | *Rabbit Polyclonal IgG* | *Abcam (ab2426)* | *1:200 in 5%BSA/TBST* |
| *α-tubulin* | *Mouse monoclonal [DM1A]* | *Abcam (ab7291)* | *1:5000 in 5%BSA/TBST* |

**Table S2**. Specifications and sources of antibodies used for immunoblotting
